# In Situ Generation of Fluorescent Copper Nanoclusters Embedded in Monolithic Eggshell Membrane: Properties and Applications

**DOI:** 10.3390/ma11101913

**Published:** 2018-10-09

**Authors:** Lu Li, Min Huang, Xianhu Liu, Dengming Sun, Congying Shao

**Affiliations:** College of Chemistry and Materials Science, Huaibei Normal University, Huaibei 235000, China; huaibeiustc@163.com (L.L.); Swordaonline@163.com (M.H.); liuxhv@126.com (X.L.); sundengming@126.com (D.S.)

**Keywords:** copper nanoclusters, fluorescence, eggshell membrane, monolithic, applications

## Abstract

Luminescent metal nanoclusters have attracted considerable research attention in recent years due to their unique properties and extensive usage in many fields. Three different synthetic routes were developed to in situ generate orange and red emitting copper nanoclusters embedded in monolithic eggshell membrane (Cu NCs@ESM) using different reducing reagents including N_2_H_4_·H_2_O, NH_2_OH·HCl and Vitamin C at room temperature for the first time. The routes are extremely facile, low-cost and versatile. The obtained Cu NCs@ESM nanocomposites exhibit excellent photostability and chemical stability, laying the foundation for various practical applications. Fluorescent surface patterning was demonstrated based on the proposed strategy easily. Significantly, the Cu NCs@ESM shows selective fluorescence quenching response to Hg^2+^ ions and good catalytic activity for methylene blue (MB) reduction degradation making it ideal as portable sensing strip and recyclable catalyst. The work provides a general strategy for the fabrication of other various monolithic nanomaterials with potential applications.

## 1. Introduction

Metal nanoclusters (NCs) consisting of several to tens of atoms have drawn considerable research interest due to their low toxicity, good biocompatibility, multifunctional surface chemistry, and excellent fluorescence behaviors such as emitting tunability, large Stokes shift, and photo stability [1,2,3,4]. The NCs have been wildly applied in bioimaging [5,6,7], sensing [8,9,10], catalysis [11,12,13,14], nanodevices [15,16,17] and many other fields [18,19]. However, to date, extensive research on fluorescent metal nanoclusters has mainly focused on the noble metal nanoclusters (especially Au and Ag NCs). The studies on tiny Cu NCs are still deficient owing to their inherent instability [17,20]. The less use of noble metals has attracted ever increasing attention due to their scarce natural resources and high cost. Researchers turned to the studies of the first transition metals such as Cu, Fe, Ni, which can be regarded as alternatives to Au and Ag. Copper is frequently used in industry because of its high conductivity, similar properties to gold and silver and especially much lower cost. Therefore, the development of various synthetic routes and extensive properties investigations of Cu NCs will be of great practical significance [21,22].

Recently, many efforts have led to some approaches for Cu NCs syntheses such as polymer and biomolecule templated methods [23,24,25,26,27,28,29,30,31], electrochemical reduction [32], sonochemical synthesis [33], microwave-assisted [34,35,36] and microemulsion technology [37,38]. However, those as-prepared Cu NCs are mostly dispersed in homogeneous solution. Agglomeration, precipitation and recycling during practical applications are the chief drawbacks of solution-dispersed fluorescent Cu NCs. Additionally, these aqueous Cu NCs might hinder their potential applications as solid state materials in chemical sensing strips, surface patterning, light-emitting devices and other fields. However, to date, there are only few reports on the syntheses of solid state Cu NCs. Zhang et al. reported the synthesis of solid fluorescent Cu NCs in supramolecular hydrogels via employing a series of bile acid derivatives as pre-gelators [39]. Qing et al. proposed the fluorescent Cu NPs templated by poly(thymine) in the agarose hydrogel [40]. Very recently, composite polymer films incorporating luminescent Cu NCs were fabricated in a hydrogel by the precursors of polyvinylpyrrolidone and poly(vinyl alcohol) by Wang et al. [41]. In the mentioned work, the solid Cu NCs were all generated based on pre-formed or simultaneously polymerized hydrogel network. Obviously, sometimes poor dispersion of NCs in hydrogel would lead to the phase segregation making it difficult to fabricate large-area, uniform, photo-stable luminescent films. Thus, developing some facile and practical strategies to fabricate novel solid state Cu NCs is a very urgent and fascinating task.

**Scheme 1:**
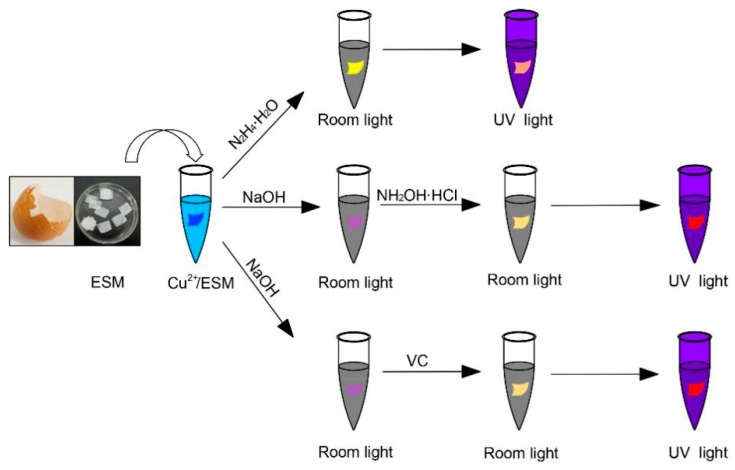
Schematic representation of different synthetic routes for the in situ generation of fluorescent copper nanoclusters embedded in monolithic eggshell membrane (Cu NCs@ESM).

Eggshell membrane (ESM), generally abandoned in daily life, has been demonstrated to be one of nature’s gifts for nanomaterial preparation [42]. ESM, a double-layer water-insoluble biomembrane with interwoven fibrous structure attached inside the hen eggshell, is composed of highly cross-linked and cystine-abundant proteins, which make ESM a good reducing agent and stabilizer in nanoscience. Importantly, the three-dimensional monolithic structure could serve as an ideal platform, providing rich sites and making sure there is uniform loading. For instance, previous research by Shao et al. demonstrated that ESM was an efficient solid state platform for generating Au and Ag NCs [43]. Also, Devi reported the formation of Ag NPs by ESM [44]. Again, Pramanik proposed the formation of fluorescent AuAg alloy nanoparticles immobilized in solid ESM [45]. Nevertheless, to the best of our knowledge, the in situ synthesis of fluorescent Cu NCs embedded in monolithic ESM hasn’t been reported yet. Additionally, the ease of oxidation of Cu (E^0^, 0.34 V), in comparison to that of Ag (E^0^, 0.80 V) and Au (E^0^, 1.50 V) makes related study remain a challenge. In this work, we established several facile routes for the in situ generation of fluorescent Cu NCs embedded in solid state ESM (Cu NCs@ESM), employing different reducing agents at room temperature for the first time (Scheme 1), and the reaction conditions and properties of obtained Cu NCs@ESM nanocomposites were investigated in detail. Theoretical and experimental studies have shown that Cu NCs possess unique size- and composition-dependent catalysis properties [1,13,14]. With many attractive features of the in situ generated Cu NCs-incorporated nanocomposite, including monolith, low cost, convenient tailoring, good stability, easy storage and transport, and large Stokes-shift, the as-prepared Cu NCs@ESM nanocomposite is expected to exhibit significant catalytic and sensing performance. Herein, their applications in visual Hg^2+^ strips sensing based on fluorescence quenching and catalysis for methylene blue reduction degradation have been demonstrated.

## 2. Materials and Methods

### 2.1. Chemicals and Materials

Copper sulfate (CuSO_4_, >99%), sodium hydroxide (NaOH, >96%), sodium borohydride (NaBH_4_, >98%), hydrazine hydrate (N_2_H_4_·H_2_O, 85%), Vitamin C (VC, >99.7%), hydroxylamine hydrochloride (NH_2_OH·HCl), mercury nitrate (Hg(NO_3_)_2_), ethanol (99.7%), and methylene blue (MB) were purchased from Sinopharm Chemical Reagent Co., Ltd (Shanghai, China). Other chemicals are all of analytical grades. All the reagents were used as received without further purification. Deionized water with a conductivity of 18.2 MΩ·cm^−1^ was purified through a Millipore water purification system and was used through the experiment.

Fresh eggs were purchased from ZhenBang supermarket near the HuaiBei Normal University (Huaibei, China). The ESMs were peeled off from the natural eggshells manually and cleaned with copious amount of deionized water carefully. The resultant ESMs were cut into proper rectangular pieces (about 1 × 1.5 cm^2^) and stored in deionized water before use.

### 2.2. Instruments

Fluorescence spectra of Cu NCs@ESMs were recorded with a FP-8300 spectrophotometer equipped with solid sample holder (Jasco, Tokyo, Japan). The photos of all samples were taken with a MeiZu mobile phone (Zhuhai, China). The fluorescent photos under 365 nm UV light were taken under a ZF-20D black-box-style UV based analyzer (YuHua, GongYi, China). Surface morphology of fluorescent Cu NCs@ESMs were observed under an Olympus IX71 fluorescence microscope (Tokyo, Japan). The X-ray photoelectron spectra (XPS) were obtained on an ULVAC-PHI X-ray photoelectron spectrometer (PHI Quantera, Tokyo, Japan) using Al K_α_ radiation. Quantitative analyses of the copper contents in Cu NCs@ESMs were performed on Perkin Elmer Opyima 7300 ICP-AES (Waltham, MA, USA). The UV-vis absorption spectra and diffuse reflectance spectra (DRS) measurements were carried out using a PGENERAL TU-1901 UV-vis spectrophotometer (Purkinje General Instrument, Beijing, China). The time-resolved fluorescence decay was investigated by time correlated single photon counting (TCSPC) technique on FLS 920 (Edinburgh Instrument, Edinburgh, UK).

### 2.3. Synthesis of Cu NCs@ESM Using N_2_H_4_·H_2_O

A cleaned piece of ESM platform was incubated in an aqueous of CuSO_4_ solution (1 mL, 50 mM) for 10 min. Then, excessive CuSO_4_ was removed, and the Cu^2+^ impregnated ESM (Cu^2+^/ESM) was transferred into 1 mL N_2_H_4_ solution (85%) to initialize the reduction. The process typically took approximately 5~7 h at ambient temperature to get strongly orange emitting Cu NCs@ESM.

### 2.4. Synthesis of Cu NCs@ESM Using NH_2_OH·HCl or VC

In a typical process, a piece of ESM was incubated in a solution of CuSO_4_ (1 mL, 50 mM) for 10 min. After that, the obtained Cu^2+^/ESM was treated with NaOH solution (1 mL, 10 M) for 20 min, and then transferred into 1 mL 1 M NH_2_OH·HCl or 2 M VC solution, immediately. The reaction took 5 h to generate intense red fluorescent Cu NCs@ESM at ambient temperature.

### 2.5. Surface Patterning on ESM Substrate

Firstly, “Cu-N-C-s” letters or the pattern of smiling face were written directly on an ESM substrate with 50 mM CuSO_4_ solution as “ink”. Then, the ESM with the impregnated letters or pattern was soaked into 10 M NaOH solution for 20 min. Finally, the treated ESM was transferred into 2 M VC solution for 5 h, and the fluorescent letters or pattern were fabricated embedded in the ESM substrate.

### 2.6. Response of Cu NCs@ESM Strips to Hg^2+^ Ions

In a typical procedure, the as-prepared monolithic Cu NCs@ESM was cut into small pieces. Then, the small pieces were added into 1 mL Hg(NO_3_)_2_ solution with different concentrations and incubated at ambient temperature. After that, the fluorescence was observed under 365 nm UV light. Meanwhile, the influence of other interferences was also investigated with the concentrations of 500 μM.

### 2.7. Catalytic Activity of Cu NCs@ESM for MB Reduction

Typically, the as-prepared Cu NCs@ESM nanocomposite was briefly thrown into 1 mL 30 μM methylene blue (MB) solution in the sunlight. The catalytic degradation of MB dye in the presence of Cu NCs@ESM was monitored by the UV-vis spectrometer.

## 3. Results and Discussion

### 3.1. Synthesis and Characterization of Cu NCs@ESM Using N_2_H_4_·H_2_O

**Figure 1:**
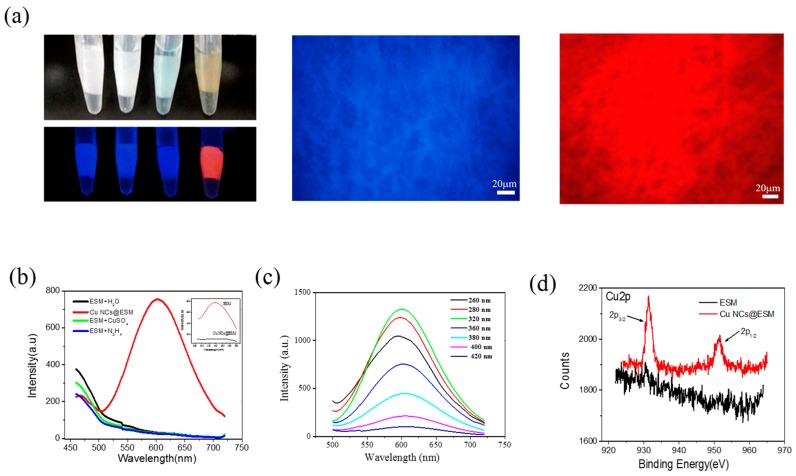
Characterization of ESMs embedded with or without CuNCs. (**a**) Room light (upper panel) and UV light (lower panel) photos of Cu NCs@ESM and controls. Samples from left to right correspond to ESM in H_2_O, ESM in N_2_H_4_·H_2_O, ESM in CuSO_4_, and Cu NCs@ESM, respectively. The fluorescence microscopy image of ESM (middle) and as-obtained Cu NCs@ESM (right) excited by 365 nm; (**b**) Fluorescence emission spectra of 1–4 samples excited by 365 nm. Inset: fluorescence spectra of ESM itself before and after loading Cu NCs in the blue light region; (**c**) Fluorescence emission spectra of Cu NCs@ESM with varying excitation wavelengths from 260 to 420 nm; (**d**) XPS spectra of the Cu NCs@ESM and ESM in the Cu 2p region.

Previous research demonstrated the formation of noble Au and Ag NCs based on the reductive ability of ESM itself in alkaline solution [43,45,46]. What about the in situ synthesis of Cu NCs employing ESM as the solid state platform? Herein, in initial experiments the ESMs were treated by these similar ways with curiosity [43] (Figures S1–S3). The results show the color of ESMs incubated with Cu^2+^ rapidly changed from blue to purplish red in alkaline solution, as described in other literatures [20,47], which indicated the formation of Cu-ESM complex. However, no fluorescent Cu NCs were further formed, even if the reaction was prolonged to several days or the reaction was allowed to proceed at elevated temperature (55 °C) employing ESM itself as the reducer in alkaline solution, which was greatly different from that of Au-ESM and Ag-ESM. Therefore, new different routes would be needed to generate fluorescent Cu NCs embedded in monolithic ESM. We then attempted to introduce an external reductant, N_2_H_4_·H_2_O, to initialize the reduction process. Typically, the ESM displayed a blue color after treating with CuSO_4_ solution for 10 min, indicating that Cu^2+^ was adsorbed onto ESM due to the coordination between Cu^2+^ and the functional groups (amines, amides, and carboxylic groups) of the ESM fibers [47]. Exhilaratingly, after the introduction of N_2_H_4_·H_2_O, the absorbed Cu^2+^ could be reduced to Cu(0) and thus induced simultaneous nucleation and size-regulating growth of fluorescent Cu NCs embedded in ESM platform. Figure 1a shows the obtained Cu NCs@ESM had a yellowish color under room light characteristic of ultrafine Cu NCs and emitted intense orange fluorescence centered at 602 nm excited with 365 nm UV light (Figure 1b). Separate control experiments were simultaneously performed by treating ESMs with N_2_H_4_·H_2_O and CuSO_4_, respectively, and no orange fluorescence was observed. Additionally, the in situ generated Cu NCs showed an excitation maximum at 317 nm (Figure S4). Besides, the excitation dependence property of Cu NCs embedded in ESM was also investigated, and no obvious excitation-dependent behavior was observed when the excitation wavelengths changed from 260 to 420 nm, indicating that the size distribution of in situ generated Cu NCs is equally uniform (Figure 1c). Fluorescence microscope images (Figure 1a) show the characteristic interwoven fibrous structure of ESM remained almost unchanged after the surface and inside loading of orange emitting Cu NCs, when compared with the natural blue emitting ESM substrate. It is noteworthy that the blue fluorescence was dramatically suppressed after the in situ generation of Cu NCs compared with the ESM itself (inset in Figure 1b). One possible reason was that the oxidation and reduction of some components in the natural ESM substrate occurred during the reaction process. On the other hand, the energy transfer between the blue emission from ESM itself and the clusters or other products was not unexpected when exited with the 365 nm UV light [43]. The content of copper in monolithic Cu NCs@ESM was determined to be 1.2% by inductively coupled plasma atomic emission spectroscopy (ICP-AES). XPS analysis also verified the existence of Cu(0) on the ESM, which showed two strong peaks at 932.2 eV and 952.2 eV in the spectrum, corresponding to the Cu2p_3/2_ and Cu2p_1/2_ of Cu(0), respectively. The absence of Cu2p3/2 satellite peak around 942 eV indicated the absence of Cu(II) in the prepared Cu NCs@ESM (Figure 1d). As we know, the 2p_3/2_ binding energy of Cu(0) is only ~0.1 eV away from that of Cu(I) species. Therefore, the valence state of the Cu in protein matrix most likely lies between 0 and +1 [13,20].

In the synthetic route, the concentrations of CuSO_4_ precursor and N_2_H_4_·H_2_O reducer played crucial roles in generating fluorescent Cu NCs. Additional experiments were performed (Figures S5 and S6) and the results show more than 5 mM Cu^2+^ and 85% N_2_H_4_·H_2_O solution (~17 M) were effective to form strongly orange emitting Cu NCs embedded in ESM after the reaction duration of 5 h at room temperature. Interestingly, when the prepared monolithic Cu NCs@ESM was always immersed in N_2_H_4_·H_2_O reaction solution until several days at 4 °C, the membrane gradually dissolved due to the alkaline hydrolysis and a homogeneous solution with brownish yellow was obtained, suggesting the immobilized Cu NCs were released into aqueous solution and dispersed homogeneously. The maximum excitation and emission peaks were observed at 337 nm and 606 nm, and the aqueous Cu NCs was excitation-independent as the parent Cu NCs@ESM (Figure S7). The large Stokes shift up to 269 nm of the Cu NCs makes it actually applicable with very low background and light scattering interference, which is ideal for bioimaging and biosensing.

### 3.2. Synthesis and Characterization of Cu NCs@ESM Using NH_2_OH·HCl

In the above case, the fluorescent Cu NCs were successfully in situ generated in ESM by externally introduced N_2_H_4_·H_2_O as the reducer. In order to develop diverse synthetic routes, other reducing agents, NaBH_4_ and NH_2_OH·HCl were also tried to prepare fluorescent Cu NCs@ESM employing the similar synthetic route. However, very weakly red emitting Cu NCs could be obtained via NaBH_4_ reduction. In particular, the reaction condition was relatively harsh, and it seemed very difficult to control a good nucleation and growth of copper clusters due to the strong reducing capacity and instability of NaBH4 itself (Figure S8). Meanwhile, no fluorescent CuNCs could be generated by NH_2_OH·HCl reduction in the case. One possible reason is that, comparable to NaBH_4_ and N_2_H_4_·H_2_O, the lower reducibility of NH_2_OH·HCl was uncapable of converting Cu(II) to Cu(0) effectively.

**Figure 2:**
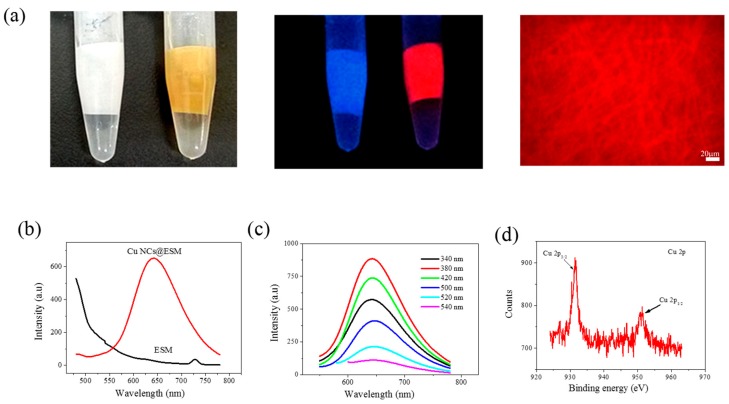
Photos and spectra of Cu NCs@ESM by NH_2_OH·HCl reduction under alkaline condition. (**a**) Photos of Cu NCs@ESM under room light (left) and 365 nm UV light (middle) along with the fluorescence microscopy image (right); (**b**) Fluorescence emission spectra of Cu NCs@ESM and ESM excited by 365 nm; (**c**) Fluorescence spectra of Cu NCs@ESM with varying excitation wavelengths from 300 to 560 nm; (**d**) XPS spectrum of Cu NCs@ESM in the Cu 2p region.

Intriguingly, it was found that the Cu^2+^/ESM treated subsequently with strong alkaline solution before throwing into NH_2_OH·HCl could be reduced to obtain red fluorescent Cu NCs effectively. In the typical procedure, ESM was briefly incubated in a CuSO_4_ solution (1 mL, 50 mΜ) for 10 min, and then the ESM immobilized with Cu^2+^ was soaked in a NaOH solution of 10 M for 30 min during which the membrane color changed to purplish red from blue indicating the formation of ESM-Cu complex [20,47], and finally transferred into 1 M NH_2_OH·HCl to initiate the reduction. As the reaction proceeded for 1 h, the color of the membrane changed from purplish red to yellow, suggesting the formation of Cu NCs embedded in ESM. Figure 2a shows the images of obtained Cu NCs@ESM by NH_2_OH·HCl reduction under alkaline environment. The Cu NCs@ESM emitted obvious red fluorescence peaked at 652 nm (Figure 2b), and the maximum excitation was 370 nm (Figure S9). The generated Cu NCs embedded in monolithic ESM have excitation-independent property (Figure 2c) suggesting the uniform size and narrow distribution of Cu NCs. XPS analysis further revealed the existence of Cu(0) embedded in ESM (Figure 2d), and the total copper content was determined to be 2.4% by ICP-AES.

As shown in Figure S10, weakly red emitting Cu NCs were generated after reaction in NH_2_OH·HCl solutions for 1~6 h, when the Cu^2+^/ESMs were treated subsequently with 500 mM~5 M NaOH aqueous solution before throwing into 1 M NH_2_OH·HCl, respectively. Even more, no fluorescence was observed during the whole reaction with a NaOH concentration lower than 500 mM. However, when the concentrations of NaOH solution reached 10~12 M, the visible red emitting Cu NCs could be in situ generated within 30 min, and the fluorescence strength reached a maximum after 5 h. Obviously, in the procedure employing NH_2_OH·HCl as reductant, the strong alkaline condition was essential for the generation of red fluorescent Cu NCs. First, in acid and neutral aqueous solution, free carboxyl and amino groups in ESM were partly dissociated into negatively charged species, incubating ESM with Cu^2+^ immediately produced Cu^2+^/ESM association by their electrostatic attraction and coordination. Subsequently, the acidity of solution was adjusted to more than pH 12 by adding NaOH and this resulted in an increase in the degree of exposure of free amino groups which could combine with Cu^2+^ easily to form a stable complex. Second, the reducing capability of tyrosine residues can be greatly improved by adjusting the reaction pH above the pKa of Tyr (~10). Hence, the reducing capability of ESM itself was not unexpected since it contains Tyr residues and possibly other residues with reduction functionality. Third, Kodali et al. reported the presence of considerable amount of cysteine-rich ESM proteins with multiple disulfide crosslink [48]. The disulfide bonds could be cleaved into free -SH under alkaline environment. And the -SH can easily combine to form stable Cu-S bond, which can stabilize the nucleated clusters. The influences of other process parameters, such as incubation time, the concentrations of CuSO_4_ precursor and NH_2_OH·HCl were also investigated systematically, which were shown in Figure S11–S13.

### 3.3. Synthesis and Characterization of Cu NCs@ESM Using VC

**Figure 3:**
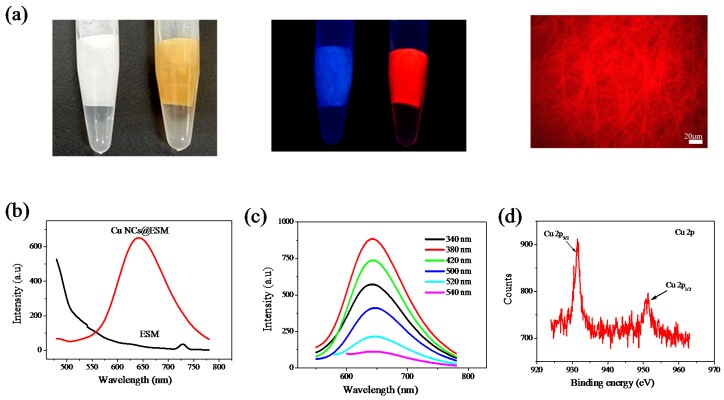
Photos and spectra of Cu NCs@ESM by VC reduction under alkaline condition. (**a**) Photos of obtained Cu NCs@ESM under room light (left) and 365 nm UV light (middle) along with the fluorescence microscopy image (right); (**b**) Fluorescence emission spectra of Cu NCs@ESM and ESM excited by 365 nm; (**c**) Fluorescence spectra of Cu NCs@ESM by varying excitation wavelengths from 340 to 570 nm; (**d**) XPS spectrum in the Cu 2p region of the resulting Cu NCs@ESM.

VC is one mild and environmentally friendly reagent, which was often used in the nanomaterials preparation instead of some severe or toxic reductants. Herein, VC was also employed to generate fluorescent Cu NCs embedded in ESM. Excitedly, it was found that VC could in situ reduce Cu^2+^ loaded on ESM into fluorescent Cu NCs under alkaline solution. The obtained Cu NCs@ESM had a yellowish color and emitted strong red fluorescence centered at 643 nm under 365 nm UV irradiation with interwoven fibrous structure (Figure 3a,b). The Cu NCs@ESM had a maximum excitation at 375 nm and the maximum emission wavelength was almost invariable when the excitation wavelengths changed from 340 to 570 nm indicating the uniform size distribution of generated NCs (Figures S14 and Figure 3c). It noteworthy that different orange and red fluorescence were observed in the developed three synthetic routes, which was confirmed by the excitation and emission spectra of Cu NCs@ESM composites fabricated by employing different reducing reagents (Figures S4, S9 and S14). Although the exact size determination of the Cu NCs embedded in the ESM was difficult, the different size of clusters could be expected in different synthetic routes due to the different clustering process of Cu atoms under different reaction environment and the size-dependent fluorescence could be achieved by varying the synthetic routes [17]. The content of copper in the obtained Cu NCs@ESM was determined to be 1.6% by ICP-AES. The generation of Cu(0) on the ESM platform was further demonstrated by the Cu2P_3/2_ and Cu2P_1/2_ peaks centered at 931.6 and 951.3 eV, respectively (Figure 3d). The time-resolved fluorescence decay of the obtained Cu NCs@ESM was simultaneously investigated by time correlated single photon counting (TCSPC) technique, and the average lifetime of generated Cu NCs was calculated to be 2.84 ns (Figure S15). In the typical alkaline condition, when the concentrations of CuSO_4_ precursor was more than 10 mM and concentrations of VC was more than 1 M, the red fluorescent Cu NCs were generated in a few minutes and reached the strongest fluorescence about 5 h (Figures S16 and S17). It has to be noted that the strong alkaline environment plays a key role in the synthetic route. When the ESM was pre-incubated in 1 mL CuSO_4_ (50 mM) for 10 min, then treated with NaOH aqueous solution of concentrations lower than 0.1 M, and finally transferred to 2 M VC solution, no fluorescent Cu NCs could be generated even with a prolonged reaction (Figure S18). This may be due to the reducing capability of ESM itself and VC was initiated effectively in strong alkaline condition to generate metal nucleation and fluorescent clusters as expected.

**Figure 4:**
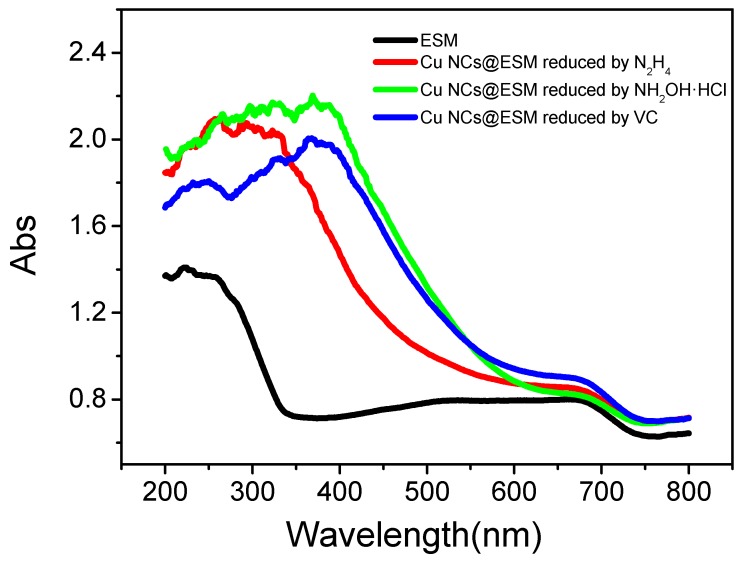
The UV-vis DRS spectra of natural ESM (black line) and as-obtained Cu NCs@ESM nanocomposites in situ generated by N_2_H_4_·H_2_O reduction (red line), NH_2_OH·HCl reduction (green line) and VC reduction (blue line), respectively.

To test the effect of copper precursors on the fluorescence of Cu NCs@ESM in the developed strategies, various copper precursors such as CuCl_2_, Cu(NO_3_)_2_, Cu(CH_3_COO)_2_, CuSO_4_ were employed in the three routes reduced by N_2_H_4_·H_2_O, NH_2_OH·HCl and VC, respectively. The results show that brightly orange and red emitting Cu NCs could be generated no matter what kind of the above four copper precursors was employed (Figure S19). Interestingly, the ESM platform, even if boiled in water, was almost equally efficient in the proposed methods for generating fluorescent Cu NCs@ESM (Figure S20). In addition, UV-Vis DRS measurement was conducted to further confirm the successful formation of Cu NCs on the ESM substrate [44]. As shown in Figure 4, there were no characteristic surface plasmon resonance (SPR) absorption of Cu nanoparticles (Cu NPs) located at ~560 nm in the spectra of Cu NCs@ESM fabricated by the three different routes, indicating the in situ generation of ultra-small Cu NCs rather than the large-sized Cu NPs [31].

**Figure 5:**
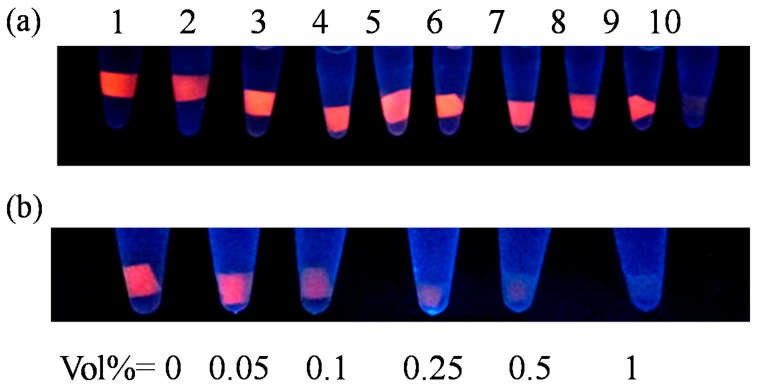
Solvent effects of Cu NCs@ESM generated by VC reduction. (**a**) the solvents are (1) water; (2) acetone; (3) formaldehyde; (4) methanol; (5) ethylene glcycol; (6) isopropanol; (7) n-butyl alcohol; (8) ethyl acetate; (9) cyclohexane; (10) ethanol, respectively; (**b**) The response of prepared Cu NCs@ESM to ethanol solution with different volume fractions, from 0 to 1% under 365 nm UV light.

It is noteworthy that the blue fluorescence was also dramatically suppressed after the generation of Cu NCs compared with the ESM itself in the NH_2_OH·HCl and VC reduction routes (Figure S21). According to the excitation spectra of the fabricated composites (Figures S9 and S14), one possible reason was that an efficient fluorescence resonance energy transfer (FRET) could happen between the ESM protein and Cu NCs, indicating a spatial proximity between the formed Cu NCs and the protein fluorophores inside the ESM, and the excellent stability of the obtained monolithic Cu NCs@ESM could be expected. On the other hand, the contribution of the oxidation or reduction of some components in the natural ESM substrate during the reaction process was not unexpected. As shown in Figure S22, the red fluorescence intensity of monolithic Cu NCs@ESM was still strong stored in wet form (dipped in water) for around 15 days and even more than a month in dried form at 4 °C in the refrigerator. Moreover, photobleaching was not observed even when the product had been continuously irradiated under 365 nm UV light for 12 h (Figure S23). Attractively, the fluorescence intensity could be maintained without any weakening even under high ionic strength conditions (1 M NaCl solution, Figure S24) for a long period of time. In addition, the Cu NCs@ESM remained very stable in the majority of organic solvents while immediate fluorescence quenching occurred in ethanol as shown in Figure 5a, and the fluorescence intensity decreased continuously with increasing volume fractions of ethanol (vol %, ethanol:H_2_O). It should be noted that a low volume fraction of 0.05% could result in an obviously distinguishable response from the background quickly (Figure 5b). The effective response of Cu NCs@ESM to ethanol content indicates the promise to develop visual sensing strips for ethanol solution and vapor conveniently based on the monolithic products. Herein, the fluorescent stability of Cu NCs embedded in ESM substrate was contributed to the protection of fibrous ESM structure and rich functional groups in the natural ESM acting as nucleation template and capping stabilizer, which avoids the aggregation and oxidation induced fluorescence quenching. In the presence of ethanol, ESM substrate was denatured mainly due to the destroy of hydrogen bonds and hydrophobicity of the composed proteins, and a looser structure was expected. Thus, the protection of ESM with Cu NCs was correspondingly weakened and the surface chemical environment of the clusters exhibited a significant change, resulting in easy oxidation of Cu NCs and fluorescence quenching of Cu NCs@ESM composite. In addition, the yellow color of the fabricated composite, which was the characteristic color of formed ultralfine Cu NCs, was found to fade obviously in the presence of ethanol (Figure S25a). Fascinatingly, the red fluorescence could be recovered when the quenched composite was replaced in a reducing agent solution such as sodium borohydride (NaBH_4_), and the yellow color reappeared, which showed the promise of the recycled ethanol sensing application (Figure S25b).

### 3.4. Surface Patterning on ESM Substrate

**Figure 6:**
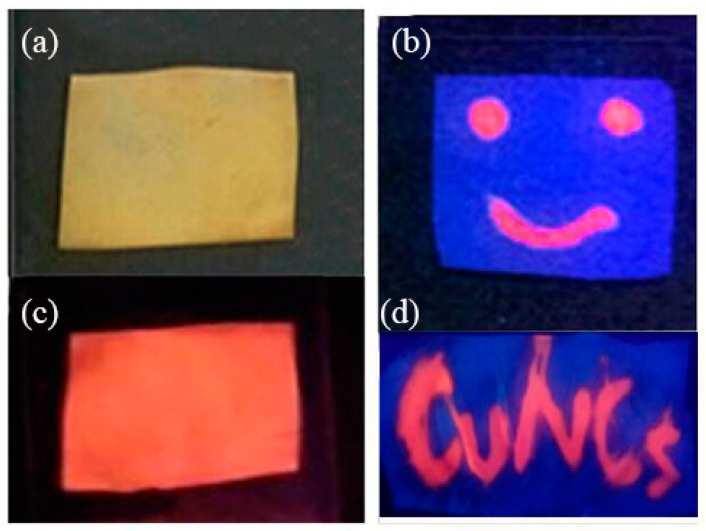
Photos of the monolithic Cu NCs@ESM fabricated by VC reduction in dried form under visible light (**a**) and 365 nm UV light (**c**); Surface fluorescence patterning of smiling face (**b**) and “Cu-N-C-s” letters (**d**) obtained by simply writing the patterning on a piece of ESM with CuSO_4_ solution as “ink” followed by NaOH and VC incubation.

Due to the excellent stability and strong fluorescence of the prepared monolithic Cu NCs@ESM in dried form, it was possible to create fluorescent patterns on the surface of ESM employing the proposed facile and green route employing VC as the reducing reagent. For example, a red fluorescent smiling face and “Cu-N-C-s” letters were obtained by simply painting them on a piece of ESM substrate with CuSO_4_ solution as “ink” followed by VC incubation under alkaline condition (Figure 6). More importantly, this work could be employed for “hidden” writing and anti-counterfeiting applications.

### 3.5. Monolithic Cu NCs@ESM Composite as Hg^2+^ Responsive Strips

**Figure 7:**
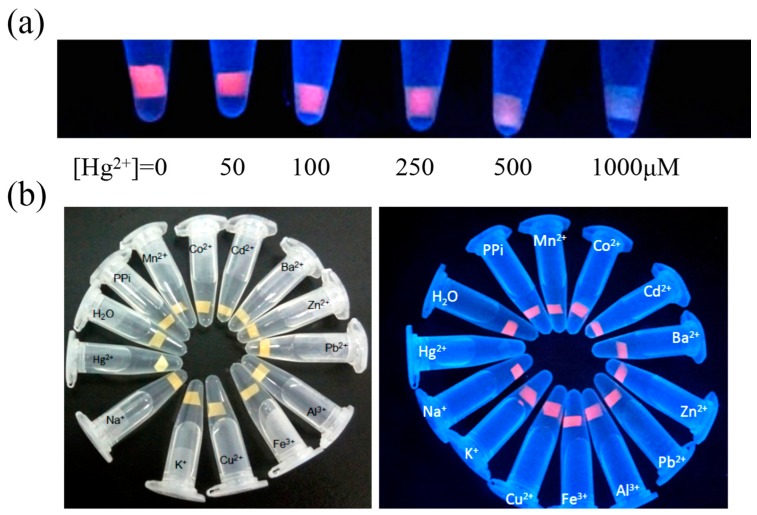
Monolithic Cu NCs@ESM as Hg^2+^ sensing strips. (**a**) Visual detection of Hg^2+^ ions photographed by daily used mobile phone at 30 min under 365 nm UV light; (**b**) Specificity test of Cu NCs@ESM strips against various other ions with the concentration of 500 μM (PPi refers to pyrophosphate ions). Photos were taken at 1 h under sunlight (left) and 365 nm UV light (right), respectively.

Developing solid state fluorescent materials for sensing purposes (a good example is pH testing paper) is of great significance with the advantages of reducing liquid handling operations (such as accurate volume measurements) and greatly lowered detection costs, which is especially useful in circumstances of in-field or POC quantitative or semi-quantitative tests. Mercury is a highly toxic heavy metal element that is widely distributed in the environment. There is a strong demand to develop low-cost and efficient techniques for Hg^2+^ detection. As a proof-of-concept demonstration, the as-prepared Cu NCs@ESM was used as visible “turn-off” strip sensor for Hg^2+^ with satisfactory sensitivity and selectivity. As shown in Figure 7b, the fluorescence of Cu NCs@ESM was quenched completely by dipping the strip in an Hg^2+^ solution for 1 h, while the fluorescence intensity was almost unchanged when incubated with other metal ions (Mn^2+^, Co^2+^, Cd^2+^, Ba^2+^, Zn^2+^, Pb^2+^, Al^3+^, Fe^3+^, Cu^2+^, K^+^, Na^+^) at the concentration of 500 μM. The current results show that the concentration of Hg^2+^ as low to 50 μM could be visibly recognized by distinguishable fluorescence quenching from the background easily (Figure 7a). The possible quenching mechanism is attributed to the high-affinity metallophilic interactions between the d^10^ centers of Hg^2+^ (5d^10^) and d^10^ centers of Cu^+^ (3d^10^) in the Cu NCs embedded in ESM. In addition, the formation of certain complexes by coordination and electrostatic attractions between Hg^2+^ and the rich functional groups (e.g., hydroxyl, carboxyl, amino and thiol) in the ESM platform was not unexpected. Overall, the surface capping-ligands and charge states of these Cu NCs were greatly changed in the presence of Hg^2+^ ions mainly due to the aforementioned interactions, which leads to the effective fluorescence quenching [49].

### 3.6. Catalytic Activity of Cu NCs@ESM for MB Reduction

**Figure 8:**
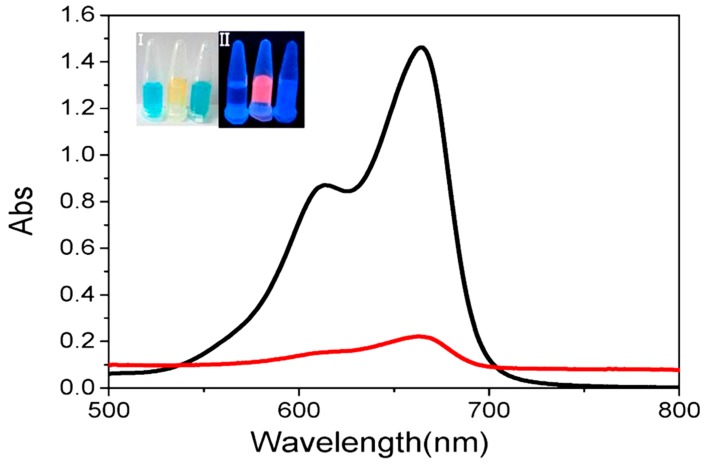
UV-vis spectra of MB solution in the absence (black line) and presence (red line) of monolithic Cu NCs@ESM reacting for 2 min. Inset: photos of MB solution (right), MB solution + Cu NCs@ESM (medium), and MB solution + ESM control (right) under room light (I) and 365 nm UV light (II).

Because of a greater proportion of high energy orbitals, ultrasmall metal NCs with usual core sizes smaller than 2 nm have been considered as active catalysts different from cousin nanoparticles and bulks [18]. Theoretical and experimental studies have shown that Cu NCs possess unique catalysis properties, and the catalytic performance of the clusters has been found strongly dependent on clusters size, composition and cluster-support interactions [1,14]. With the attractive features of the in situ generated Cu NCs@ESM nanocomposite, such as monolith, convenient operation, good stability, easy storage and transport, their potential utility as low-cost and recyclable catalyst was expected. Specifically, to examine the catalytic activity of the product, the reduction degradation of methylene blue (MB) dye was investigated as model reaction [14,18,39,50]. As shown by the inset in Figure 8, the original blue color of MB solution immediately faded after the monolithic Cu NCs@ESM was thrown into the solution due to the formation of colorless product, leucomethylene blue (LMB). However, the blue color was almost unchanged when the serial ESM controls without Cu NCs incorporated were added into the MB solutions and incubated even for a longer time, indicating that the observed catalytic performance of Cu NCs@ESM nanocomposite was really contributed to the incorporated Cu NCs, similar to that of reported Cu NCs prepared by wet chemistry methods (Figure S26a). The UV-vis spectrometer was used to optically monitor the catalyzed process. The featured maximum absorption peak at 662 nm of original MB dye was found to be quickly and dramatically decreased within 2 min, which suggests that the Cu NCs@ESM could serve as an efficient catalyst for the reduction degradation of MB without the need of either additional reducing agents or UV irradiation. Significantly, the strong fluorescence of Cu NCs@ESM was still maintained after the reduction degradation (inset in Figure 8 and Figure S26b), and the catalytic reaction could be cycled 4~5 times just by removing out and throwing into facilely, benefiting from the rigid solid monolithic characteristics of the ESM platform.

## 4. Conclusions

In summary, in situ generation of orange/red emitting copper nanoclusters embedded in monolithic eggshell membrane (Cu NCs@ESM) was successfully demonstrated in this work. To the best of our knowledge, this is the first report on the fabrication of fluorescent Cu NCs employing natural ESM as a platform, and three different synthetic routes were developed using various reducing reagents, including N_2_H_4_·H_2_O, NH_2_OH·HCl and Vitamin C (VC) under different reaction conditions. The obtained Cu NCs@ESM nanocomposites show excellent photo and chemical stability. Significantly, the routes are extremely low cost, green, facile, and versatile. Syntheses of other monolithic nanoclusters, nanoparticles and quantum dots are going on well in our laboratory employing the similar strategy based on ESM or even silkworm cocoon as the solid platforms. In addition, the selective fluorescence quenching response of Cu NCs@ESM to Hg^2+^ ions and its good catalytic activity for MB reduction degradation makes it ideal as an inexpensive sensing strip and recyclable catalyst. We believe that it would serve as a general strategy for the fabrication of various monolithic nanomaterials with potential applications in the fields of chemical sensing, heterogeneous catalysis, antibacterial nanomaterial, surface enhanced Raman scattering interface, fluorescent surface patterning, nanodevices and so on.

## Supplementary Materials

The following are available online at http://www.mdpi.com/1996-1944/11/10/1913/s1, Figure S1: Photographs of ESMs with Cu^2+^ incubated in alkaline solutions. ESMs were pre-incubated in 1 mL 50 mM CuSO_4_ solutions for 10 min, and then soaked in 0, 0.0001, 0.0005, 0.001, 0.01, 0.1, 0.5, 1 and 5 M NaOH solutions (from left to right), respectively at room temperature. Photographs of the samples were taken under room light (a) and 365 nm UV light (b) at different reaction time; Figure S2: Photographs of ESMs with Cu^2+^ under 365 nm UV irradiation. ESMs were pre-incubated in 0, 0.5, 1, 10, 50, 100, 250, 500, 750 and 1000 mM CuSO_4_ solutions (from left to right), respectively for 10 min, and then subjected to 365 nm UV irradiation to initialize the reduction process. Photos of the samples were taken under 365 nm UV light (a) and room light (b) at different reactions time; Figure S3: Photographs of ESMs with Cu^2+^ in alkaline solutions irradiated by 365 nm UV light. ESMs were pre-incubated in 50 mM CuSO_4_ solutions for 10 min, and then transferred into NaOH solutions with various concentrations (0, 1, 10, 50, 100, 250, 500, 1000, 5000, 10,000 mM, from left to right) respectively, and then subjected to UV irradiation at 365 nm. Photos of the samples were taken under 365 nm UV light (a) and room light (b) at different reactions time; Figure S4: Fluorescence excitation and emission spectra of Cu NCs@ESM reduced by N_2_H_4_·H_2_O. The maximum excitation and emission wavelengths were 317 nm and 601 nm, respectively; Figure S5: (a) Photos of the samples under UV light corresponding to various reaction times and a series of CuSO_4_ concentrations. ESMs were pre-incubated in CuSO_4_ solutions of different concentrations: 0, 0.1, 0.5, 1, 5, 10, 30 and 50 mM (from left to right) for 10 min, and then transferred to 85% N_2_H_4_·H_2_O solution to initialize the reduction reactions. (b) Room light photos of the samples after reaction of 9 h; Figure S6: (a) Fluorescence evolution of Cu NCs@ESMs reduced by N_2_H_4_·H_2_O under 365 nm UV light. ESMs were pre-incubated in CuSO_4_ solutions (1 mL, 50 mM) for 10 min, and then transferred to N_2_H_4_ solutions with various concentrations: 0, 0.05, 0.1, 0.5, 1, 5, 10, 17.49 M (from left to right) to initialize the reaction process. (b) Room light photo of the samples after reaction of 9 h; Figure S7: (a) The excitation and emmission spectra of the obtained aqueous Cu NCs solution. Inset: Photos of the Cu NCs solution under visible (left) and UV light (right). The maximum excitation and emission wavelengths were 337 nm and 606 nm, respectively. (b) Emission spectra of Cu NCs solution with varying excitation wavelengths from 300 nm to 370 nm; Figure S8: The ESMs were pre-incubated in 2, 20, 40 mM CuSO_4_ solutions, respectively, for 20 min, and then transferred to NaBH_4_ solutions of various concentrations (0, 0.5, 1, 5, 10, 50, 80 mM) to initialize the reduction. Photos of the samples were taken at room light (left) and under 365 nm UV illumination (right) after the reactions had proceeded 6 h; Figure S9: Fluorescence emission and excitation spectra of Cu NCs@ESM via NH_2_OH·HCl reduction. The maximum excitation and emission wavelengths were 370 nm and 652 nm, respectively; Figure S10: Influence of NaOH concentration on the preparation of Cu NCs@ESM at room temperature. An ESM was incubated with 1 mL, 50 mM CuSO_4_ for 10 min, and then transferred to a NaOH solution at a series of concentrations (0, 0.0005, 0.01, 0.05, 0.1, 0.5, 1, 5, 10, 12 M) for 30 min, and subsequently soaked in 1 mL 1 M NH_2_OH·HCl solution. Photos of the samples were taken under 365 nm UV light. (b) ESMs incubated in NaOH solution with different concentrations (left); ESMs incubated in NaOH with different concentrations for 30 min and then thrown into 1 mL 1 M NH_2_OH·HCl solution (right); Figure S11: Influence of incubation time of the Cu^2+^/ESM with NaOH solution on the preparation of Cu NCs@ESM. Each ESM was pre-incubated in 1 mL 50 mM CuSO_4_ solution for 10 min, and then treated with 1 mL NaOH solution (10 M) for different time (60, 30, 20, 10, 5, 0 min, from left to right tubes), and finally transferred to 1 mL NH_2_OH·HCl solution (1 M). (a) and (b) are photos of the samples taken under UV light (365 nm) and room light after reaction for 5 h. It was shown that 10 min was enough for the incubation process; Figure S12: (a) Fluorescence evolution of Cu NCs@ESMs reduced by NH_2_OH·HCl solutions under 365 nm UV light with different CuSO_4_ concentrations. ESMs were pre-incubated in 1 mL CuSO_4_ of different concentrations (0, 0.5, 1, 10, 20, 50, 60, 80, 100 mM) for 10 min, and then transferred to a 10 M NaOH solution for 30 min, and subsequently soaked in 1 mL 1 M NH_2_OH·HCl solution. (b) Room light photo of the samples after reactions of 7 h. The optimized concentration of CuSO_4_ solution was 50 mM; Figure S13: Influence of NH_2_OH·HCl concentration on the generation of the Cu NCs@ESM at room temperature. A piece of ESM was incubated with 1 mL, 50 mM CuSO_4_ for 10 min, and then transferred to 10 M NaOH solution for 30 min, and finally soaked in NH_2_OH·HCl solution at a series of concentrations (0, 0.01, 0.05, 0.1, 0.3, 0.5, 1, 3, 5 M, from left to right tubes). The optimized concentration of NH_2_OH·HCl was 1 M; Figure S14: Fluorescence emission and excitation spectra of Cu NCs@ESM via VC reduction. The maximum excitation and emission wavelengths were 375 nm and 642 nm, respectively; Figure S15: PL decay profile (λ_em_ = 642 nm) of Cu NCs@ESM reduced by VC under alkaline condition at the excitation of 373 nm. The average lifetime of generated Cu NCs was calculated to be 2.84 ns; Figure S16: Influence of CuSO_4_ concentration on the generation of Cu NCs@ESM reduced by VC at room temperature. (a) ESMs were pre-incubated in CuSO_4_ with various concentrations (0, 0.1, 0.5, 1, 5, 10, 20, 50 mM, from left to right tubes) for 10 min, then treated with 10 M NaOH solution for 30 min, and finally transferred to 2 M VC solution to initiate the nucleations. (b) Photoes of the samples at 0 min and after reactions of 7 h under room light; Figure S17: Influence of VC concentration on the preparation of Cu NCs@ESM at room temperature. (a) ESMs were pre-incubated in 1 mL CuSO_4_ (50 mM) for 10 min, then treated with 10 M NaOH solution for 30 min, and finally transferred to VC solutions with various concentrations (0, 0.001, 0.01, 0.05, 1, 1.5, 2 M, from left to right tubes). (b) Photoes of the samples at 0 min and after reactions of 7 h under room light; Figure S18: Influence of NaOH concentration on the generation of Cu NCs@ESM reduced by VC at room temperature. (a) ESMs were pre-incubated in 1 mL CuSO_4_ (50 mM) for 10 min, then treated with NaOH aqueous solution of different concentrations (0, 0.001, 0.01, 0.1, 0.5, 1, 5, 10, 15 M, from left to right tubes) for 30 min, and finally transferred to 2 M VC solution. (b) Photos of the samples at 0 min and after reactions of 7 h under room light. (b) ESMs incubated in NaOH solutions with different concentrations (left); ESMs incubated in NaOH solutions with different concentrations for 30 min and then thrown into 1 mL 2 M VC solution (right); Figure S19: The effect of different copper precursors on the generation of fluorescent Cu NCs@ESM in the different three strategies. The various copper precursors such as CuCl_2_, Cu(NO_3_)_2_, Cu(CH_3_COO)_2_, CuSO_4_ were employed (from left to right tubes) in the strategies reduced by N_2_H_4_·H_2_O (upper panels), NH_2_OH·HCl (middle panels) and VC (lower panels). Photos of the samples under room light (left) and 365 nm UV light (right), respectively; Figure S20: Photos of obtained Cu NCs@ESM generated by the three different synthetic routes employing boiled ESM platform under room light (a) and 365 nm UV light (b); Figure S21: The fluorescence emissions (λ_ex_ = 365 nm) of ESM platforms from natural ESM (black line) and ESMs embedded with fluorescent Cu NCs in situ generated by N_2_H_4_·H_2_O reduction (red line), NH_2_OH·HCl reduction (blue line) and VC reduction (purple line), respectively; Figure S22: The stability of monolithic Cu NCs@ESM generated by VC reduction under alkaline condition stored in wet (a) and dried (b) forms stored at 4 °C in the refrigerator; Figure S23: Photostability of the as-prepared Cu NCs@ESM evaluated by placing it in quartz cuvette under the 365 nm UV light irradiation from a 6 W mercury lamp with a distance of about 1 cm to the lamp tube. Photos were taken after continuous irradiation of 0, 0.5, 1, 3, 5, 7, 12 h respectively (from left to right); Figure S24: Salt tolerance of prepared Cu NCs@ESM in NaCl aqueous solutions with different concentrations (0, 0.2, 0.4, 0.6, 0.8, 1.0 M, from left to right); Figure S25: (a) The photos and fluorescence emission spectra of Cu NCs@ESM in the absence (1) and presence (2) of ethanol under room light (left) and the 365 nm UV light (right). (b) The fluorescence recovery experiment of quenched Cu NCs@ESM composite by NaBH_4_ treatment. The quenched Cu NCs@ESMs were incubated with NaBH_4_ aqueous solutions with different concentrations (500, 250, 125, 50, 25, 10, 5, 2.5 mM, from 3 to 10) to regenerate Cu NCs at room temperature for 2 h; Figure S26: (a) The photo of MB aqueous solutions by adding serial control ESMs treated with the same way, respectively. (From 1 to 7: ESM only, ESM-NaOH, ESM-CuSO_4_, ESM-CuSO_4_-NaOH, ESM-CuSO_4_-VC, ESM-NaOH-VC, ESM-VC). (b) The fluorescence emission spectra of Cu NCs@ESM composite before and after catalysis excited by 365 nm.
