# Supplementary material for: In Situ Generation of Fluorescent Copper Nanoclusters Embedded in Monolithic Eggshell Membrane: Properties and Applications
Source: Materials (Basel). 2018 Oct 9;11(10):1913. doi: 10.3390/ma11101913 (PMC6213854; doi:10.3390/ma11101913)
Supplement: Supplementary file 1 [file materials-11-01913-s001.pdf]

materials

Article

In Situ Generation of Fluorescent Copper Nanoclusters Embedded in Monolithic Eggshell Membrane: Properties and Applications

Lu Li, Min Huang, Xianhu Liu, Dengming Sun, and Congying Shao *

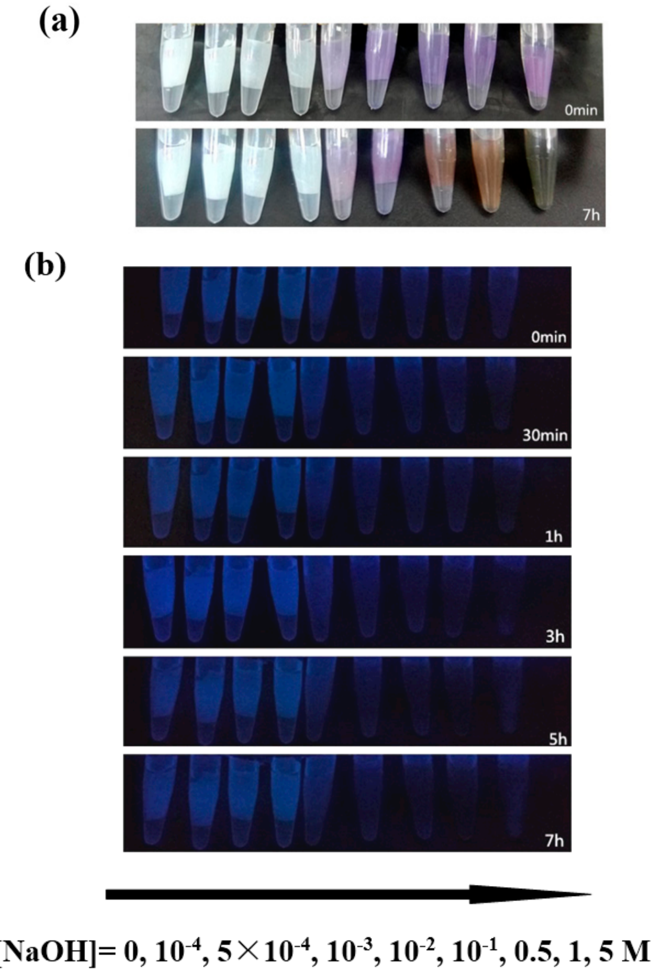

Figure S1. Photographs of ESMs with Cu^{2+} incubated in alkaline solutions. ESMs were pre-incubated in 1 mL 50 mM $CuSO_4$ solutions for 10 min, and then soaked in 0, 0.0001, 0.0005, 0.001, 0.01, 0.1, 0.5, 1 and 5 M NaOH solutions (from left to right), respectively at room temperature. Photographs of the samples were taken under room light (**a**) and 365 nm UV light (**b**) at different reaction time.

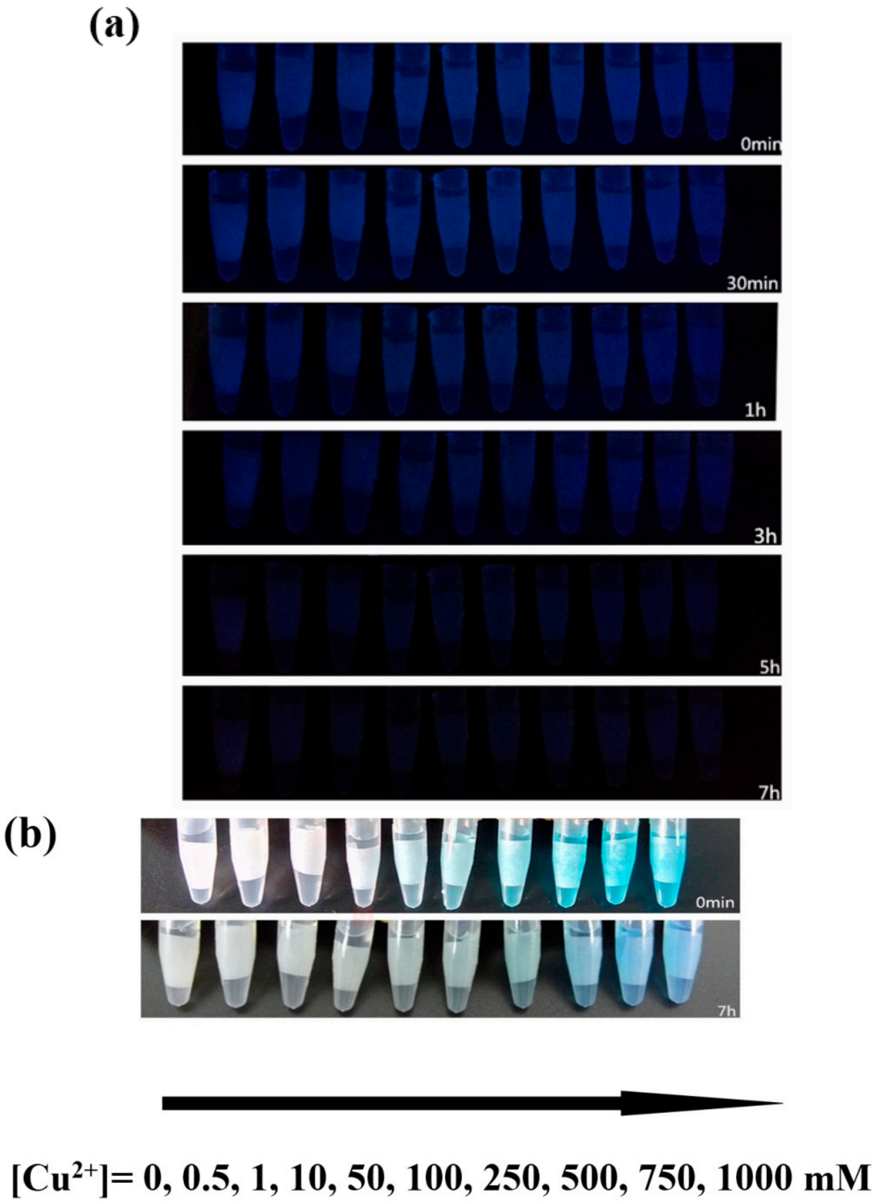

Figure S2. Photographs of ESMs with Cu^{2+} under 365 nm UV irradiation. ESMs were pre-incubated in 0, 0.5, 1,10, 50, 100, 250, 500, 750 and 1000 mM $CuSO_4$ solutions (from left to right), respectively for 10 min, and then subjected to 365 nm UV irradiation to initialize the reduction process. Photos of the samples were taken under 365 nm UV light (**a**) and room light (**b**) at different reactions time.

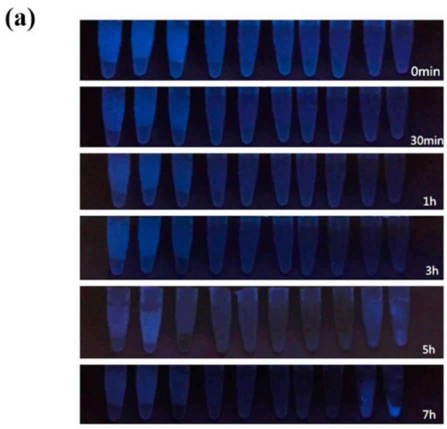

(b)

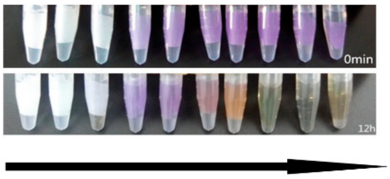

[NaOH]= 0, 1, 10, 50, 100, 250, 500, 1000, 5000, 10000 mM

Figure S3. Photographs of ESMs with Cu^{2+} in alkaline solutions irradiated by 365 nm UV light. ESMs were pre-incubated in 50 mM $CuSO_4$ solutions for 10 min , and then transferred into NaOH solutions with various concentrations (0, 1, 10, 50, 100, 250, 500, 1000, 5000, 10000 mM, from left to right) respectively, and then subjected to UV irradiation at 365nm. Photos of the samples were taken under 365 nm UV light (**a**) and room light (**b**) at different reactions time.

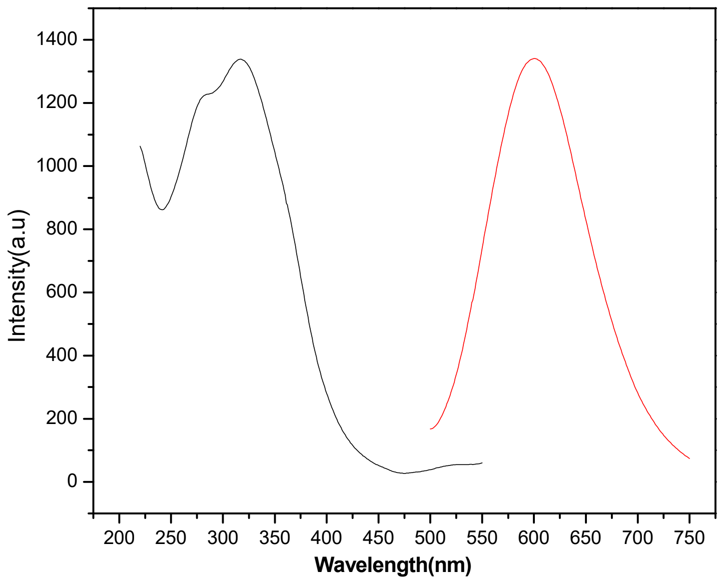

Figure S4. Fluorescence excitation and emission spectra of Cu NCs@ESM reduced by $N_2H_4{\cdot}H_2O$. The maximum excitation and emission wavelengths were 317 nm and 601 nm, respectively.

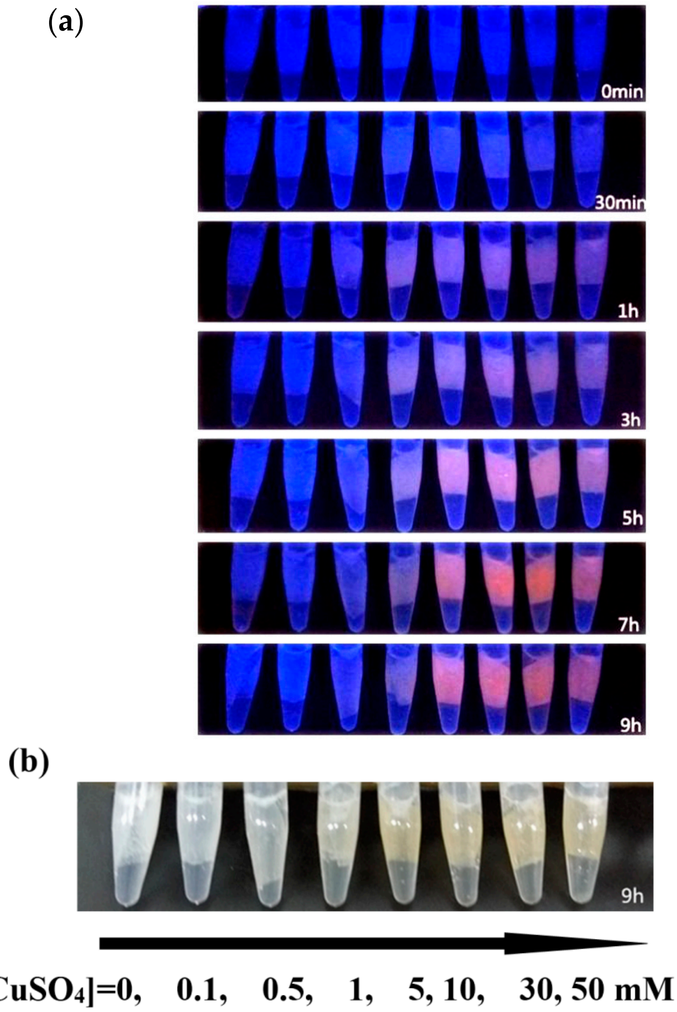

Figure S5. (**a**) Photos of the samples under UV light corresponding to various reaction times and a series of $CuSO_4$ concentrations. ESMs were pre-incubated in $CuSO_4$ solutions of different concentrations: 0, 0.1, 0.5, 1, 5, 10, 30 and 50 mM (from left to right) for 10 min, and then transferred to 85% $N_2H_4{\cdot}H_2O$ solution to initialize the reduction reactions. (**b**) Room light photos of the samples after reaction of 9 hours.

(a)

(b)

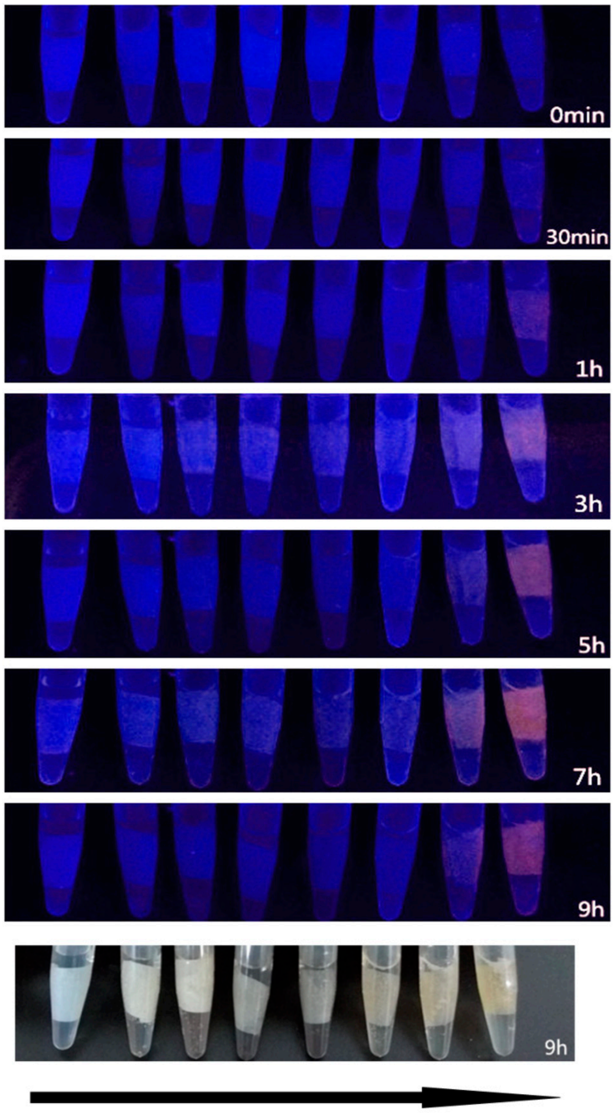

[$N_2H_4 \cdot H_2O$]=0, 0.05, 0.1, 0.5, 1, 5, 10, 17.49 M

Figure S6. (**a**) Fluorescence evolution of Cu NCs@ESMs reduced by $N_2H_4 \cdot H_2O$ under 365 nm UV light. ESMs were pre-incubated in $CuSO_4$ solutions (1mL, 50 mM) for 10min, and then transferred to N_2H_4 solutions with various concentrations: 0, 0.05, 0.1, 0.5, 1, 5, 10, 17.49 M (from left to right) to initialize the reaction process. (**b**) Room light photo of the samples after reaction of 9 hours.

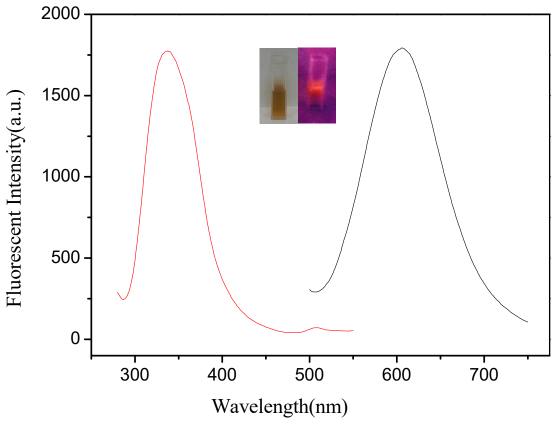

(**a**)

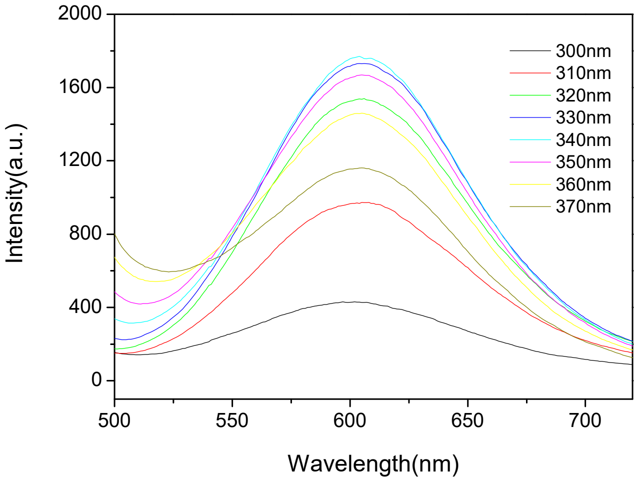

(**b**)

Figure S7. (**a**) The excitation and emmission spectra of the obtained aqueous Cu NCs solution. Inset: Photos of the Cu NCs solution under visible (left) and UV light (right). The maximum excitation and emission wavelengths were 337 nm and 606 nm, respectively. (**b**) Emission spectra of Cu NCs solution with varying excitation wavelengths from 300 nm to 370 nm.

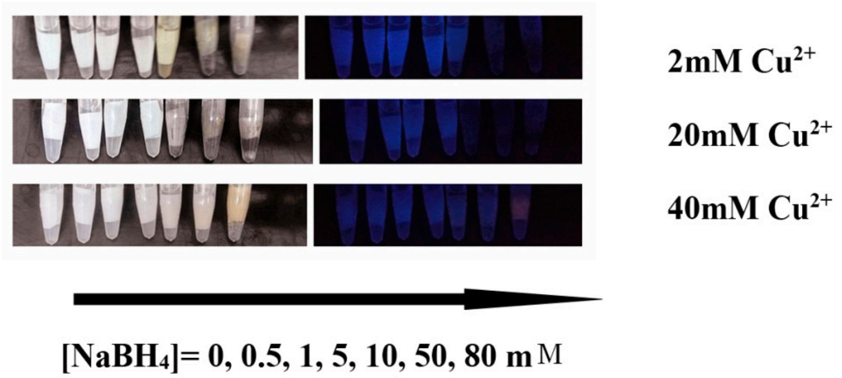

Figure S8. The ESMs were pre-incubated in 2, 20, 40 mM $CuSO_4$ solutions, respectively, for 20 min, and then transferred to $NaBH_4$ solutions of various concentrations (0, 0.5, 1, 5, 10, 50, 80 mM) to initialize the reduction. Photos of the samples were taken at room light (left) and under 365 nm UV illumination (right) after the reactions had proceeded 6 hours.

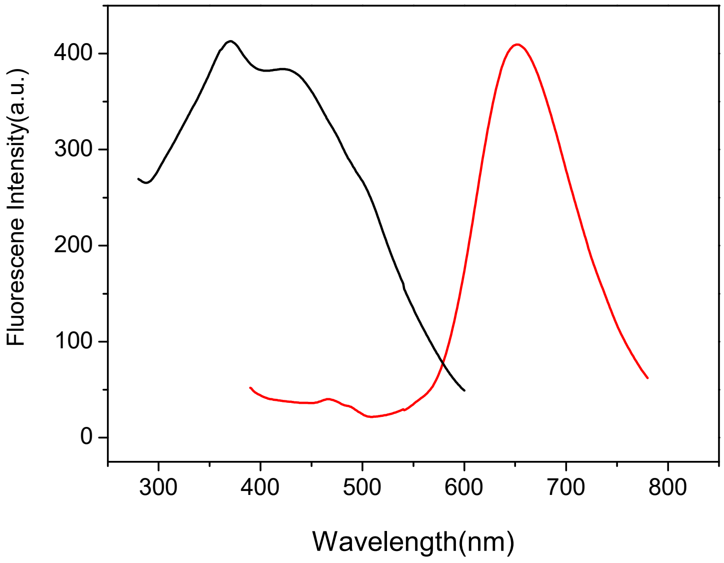

Figure S9. Fluorescence emission and excitation spectra of Cu NCs@ESM via $NH_2OH{\cdot}HCl$ reduction. The maximum excitation and emission wavelengths were 370 nm and 652 nm, respectively.

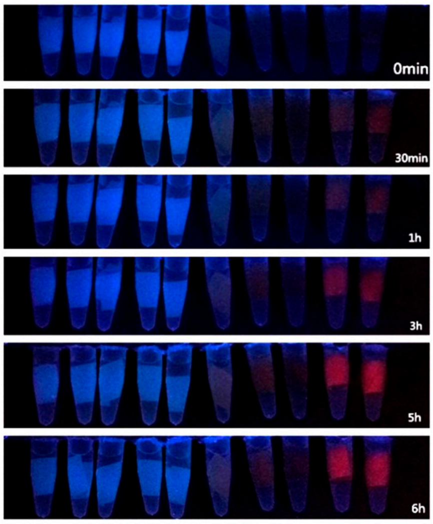

(**a**)

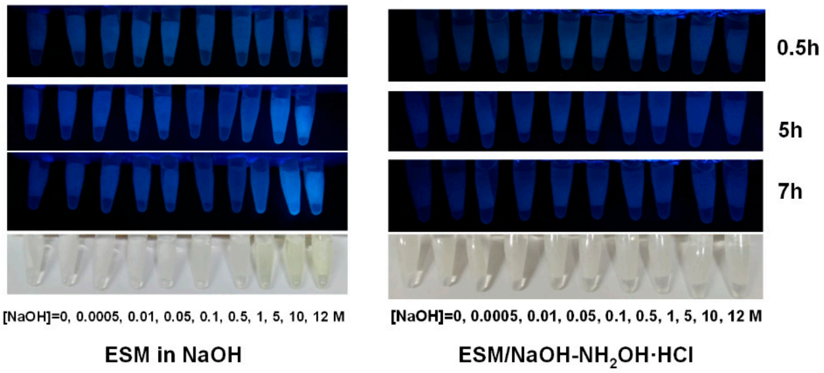

(**b**)

Figure S10. (**a**) Influence of NaOH concentration on the preparation of Cu NCs@ESM at room temperature. An ESM was incubated with 1 mL, 50 mM $CuSO_4$ for 10 min ,and then transferred to a NaOH solution at a series of concentrations (0, 0.0005, 0.01, 0.05, 0.1, 0.5, 1, 5, 10, 12 M) for 30 min, and subsequently soaked in 1 mL 1 M $NH_2OH{\cdot}HCl$ solution. Photos of the samples were taken under 365 nm UV light. (**b**) ESMs incubated in NaOH solution with different concentrations (left); ESMs incubated in NaOH with different concentrations for 30 min and then thrown into 1 mL 1 M $NH_2OH{\cdot}HCl$ solution (right).

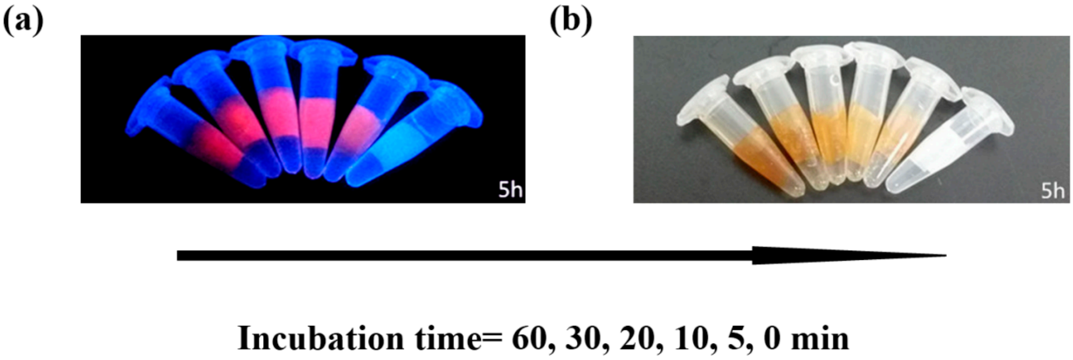

Figure S11. Influence of incubation time of the Cu^{2+}/ESM with NaOH solution on the preparation of Cu NCs@ESM. Each ESM was pre-incubated in 1 mL 50 mM $CuSO_4$ solution for 10min, and then treated with 1 mL NaOH solution (10 M) for different time (60, 30, 20, 10, 5, 0min, from left to right

tubes), and finally transferred to 1 mL $NH_2OH{\cdot}HCl$ solution (1 M). (**a**) and (**b**) are photos of the samples taken under UV light (365 nm) and room light after reaction for 5 hours. It was shown that 10 min was enough for the incubation process.

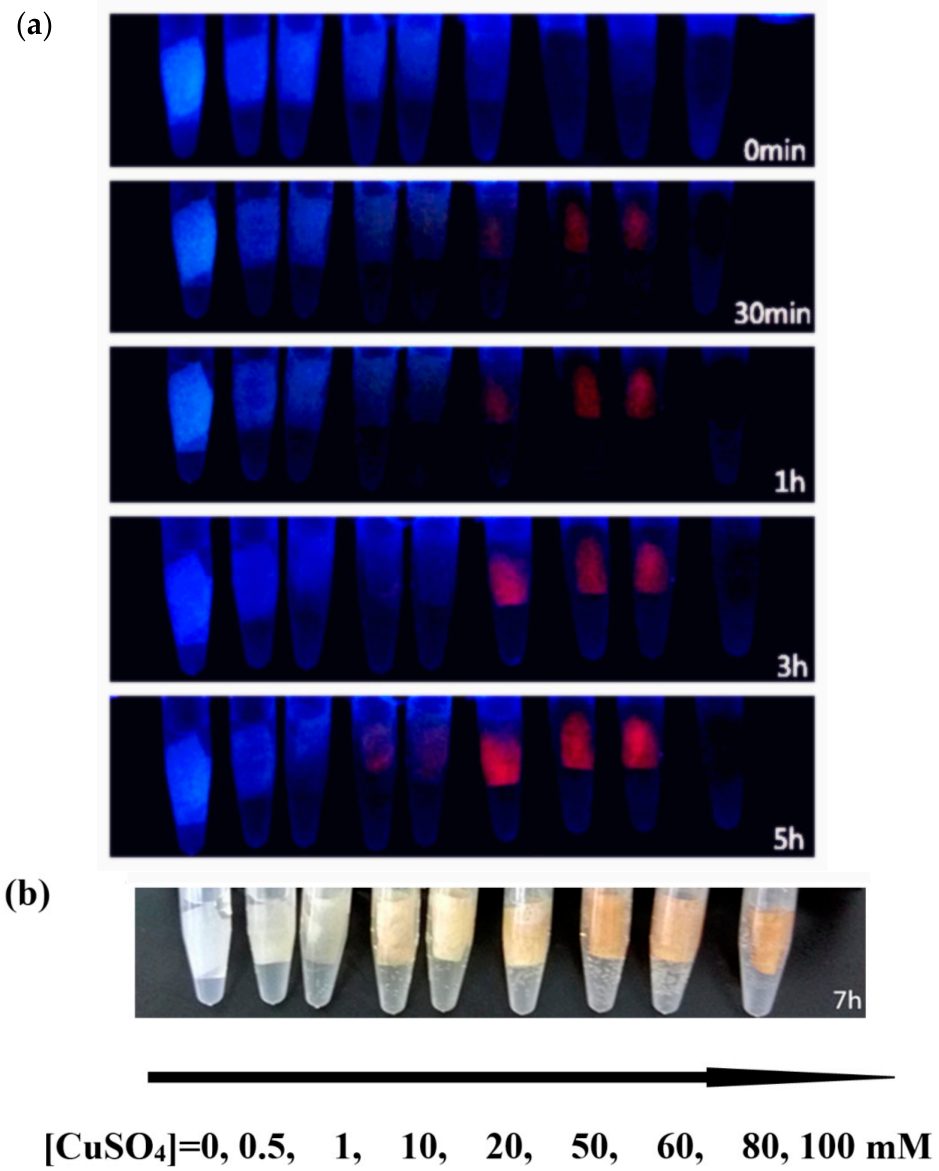

Figure S12. (**a**) Fluorescence evolution of Cu NCs@ESMs reduced by $NH_2OH{\cdot}HCl$ solutions under 365 nm UV light with different $CuSO_4$ concentrations. ESMs were pre-incubated in 1 mL $CuSO_4$ of different concentrations (0, 0.5, 1, 10, 20, 50, 60, 80, 100 mM) for 10min, and then transferred to a 10 M NaOH solution for 30 min, and subsequently soaked in 1 mL 1 M $NH_2OH{\cdot}HCl$ solution. (**b**)Room light photo of the samples after reactions of 7 hours. The optimized concentration of $CuSO_4$ solution was 50 mM.

(**a**)

(**b**)

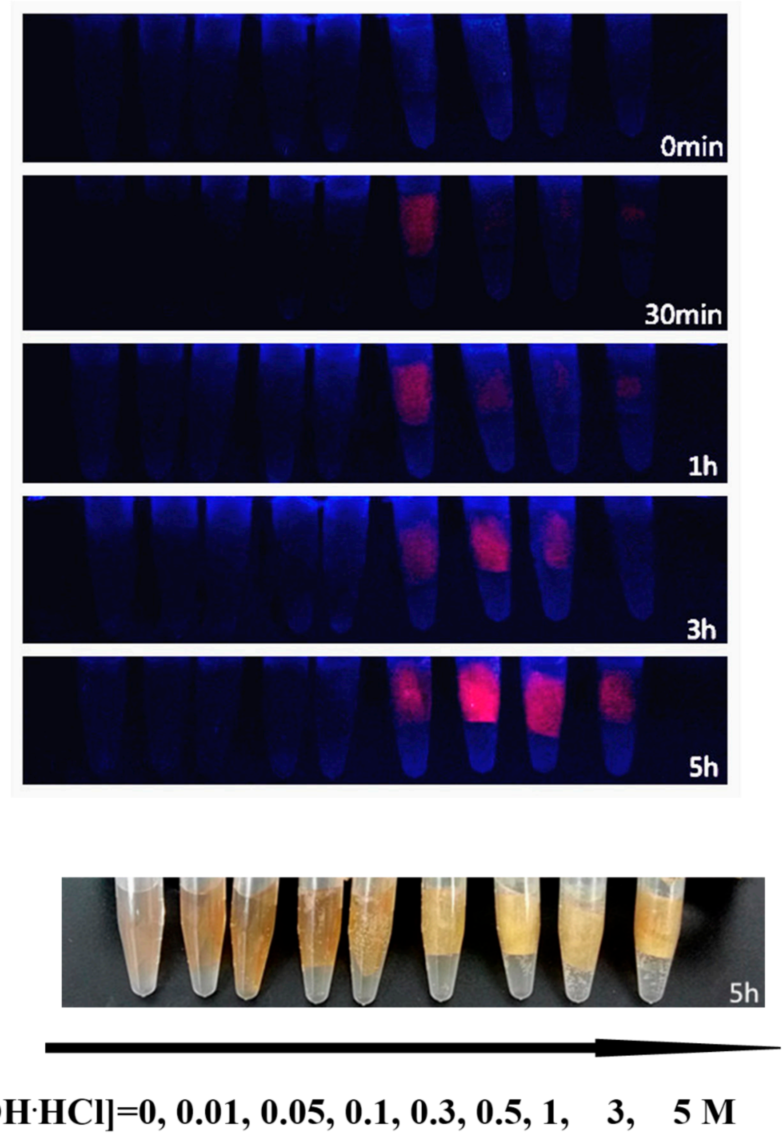

Figure S13. Influence of $NH_2OH{\cdot}HCl$ concentration on the generation of the Cu NCs@ESM at room temperature. A piece of ESM was incubated with 1 mL, 50 mM $CuSO_4$ for 10 min, and then transferred to 10 M NaOH solution for 30 min, and finally soaked in $NH_2OH{\cdot}HCl$ solution at a series of concentrations (0, 0.01, 0.05, 0.1, 0.3, 0.5, 1, 3, 5 M, from left to right tubes). The optimized concentration of $NH_2OH{\cdot}HCl$ was 1 M. (**a**) Fluorescence evolution of Cu NCs@ESMs reduced by $NH_2OH{\cdot}HCl$ photoed under 365 nm UV light with different $NH_2OH{\cdot}HCl$ concentrations. (**b**) Room light photo of the samples after reactions of 5 hours.

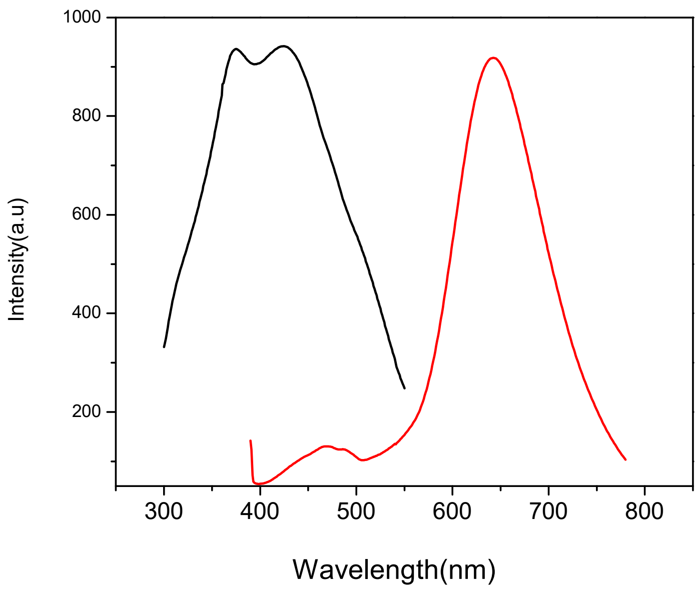

Figure S14. Fluorescence emission and excitation spectra of Cu NCs@ESM via VC reduction. The maximum excitation and emission wavelengths were 375 nm and 642 nm, respectively.

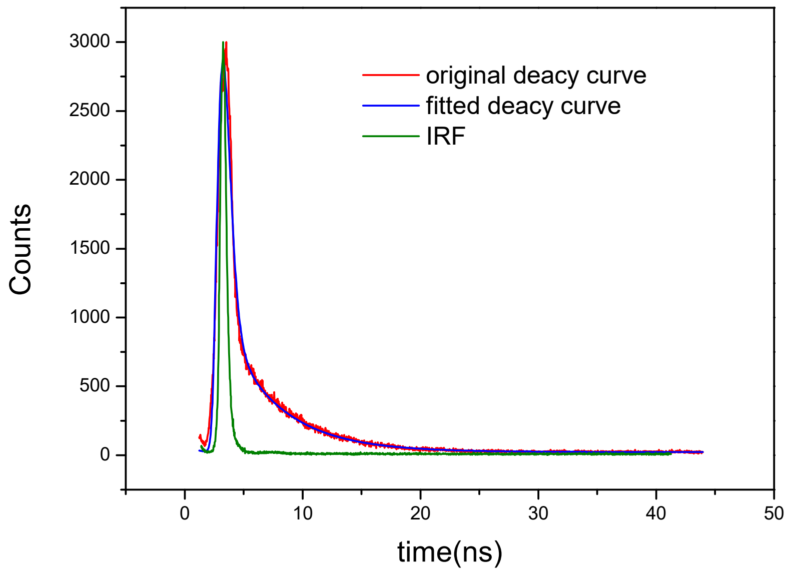

	B_i	ΔB_i	f_i (%)	Δf_i (%)	τ_i (ns)	$\Delta\tau_i$ (ns)
1	0.0564	0.0013	41.904	6.702	0.434	0.060
2	0.0074	0.0001	58.096	0.860	4.581	0.002

Note: The fluorescence decay curves was fitted based on multiple exponential function, and the average lifetime τ was calculated according to the following equation: $\tau = \frac{\sum B_n \tau_n^2}{B_n \tau_n}$ (n=1,2,3……)

Figure S15. PL decay profile (λ_{em}=642 nm) of Cu NCs@ESM reduced by VC under alkaline condition at the excitation of 373 nm. The average lifetime of generated Cu NCs was calculated to be 2.84 ns.

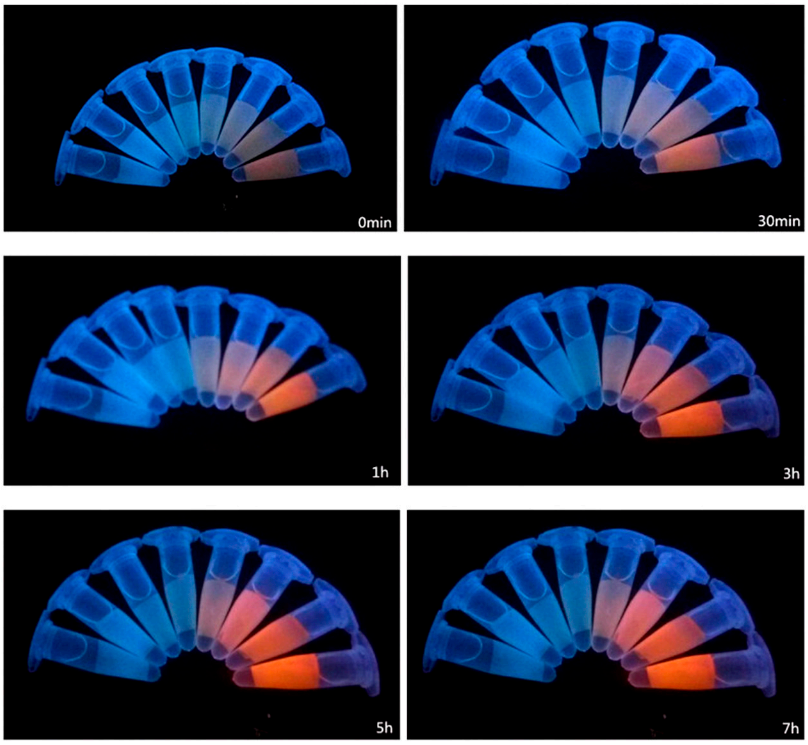

(**a**)

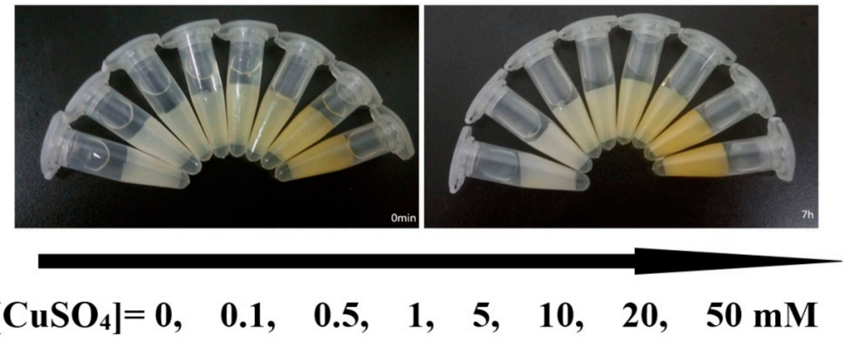

(**b**)

Figure S16. Influence of $CuSO_4$ concentration on the generation of Cu NCs@ESM reduced by VC at room temperature. (**a**) ESMs were pre-incubated in $CuSO_4$ with various concentrations (0, 0.1, 0.5, 1, 5, 10, 20, 50 mM, from left to right tubes) for 10 min, then treated with 10 M NaOH solution for 30 min, and finally transferred to 2 M VC solution to initiate the nucleations. (**b**) Photoes of the samples at 0min and after reactions of 7 hours under room light.

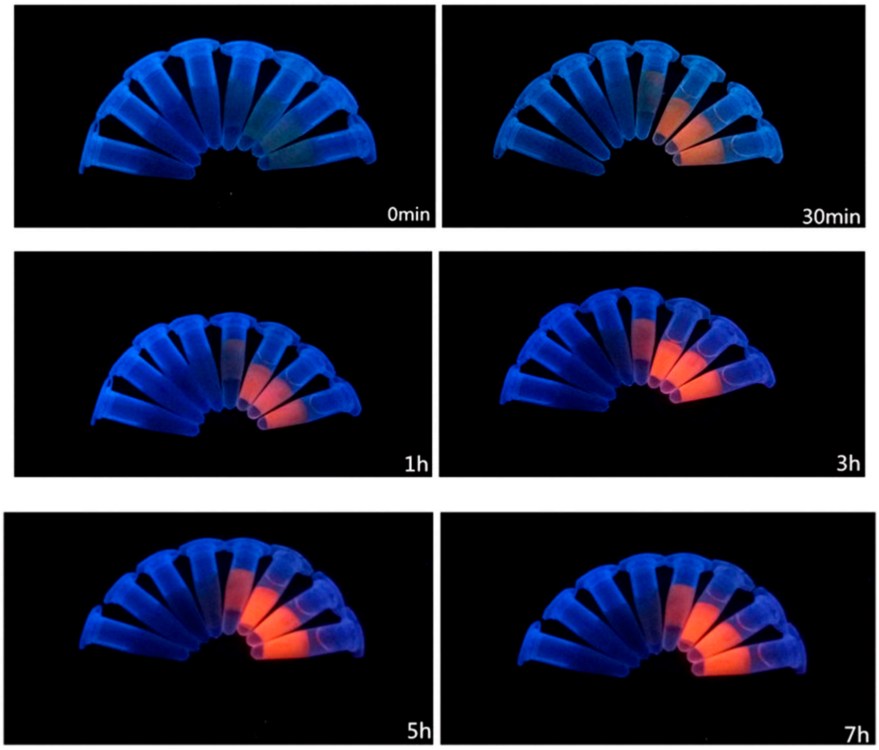

(**a**)

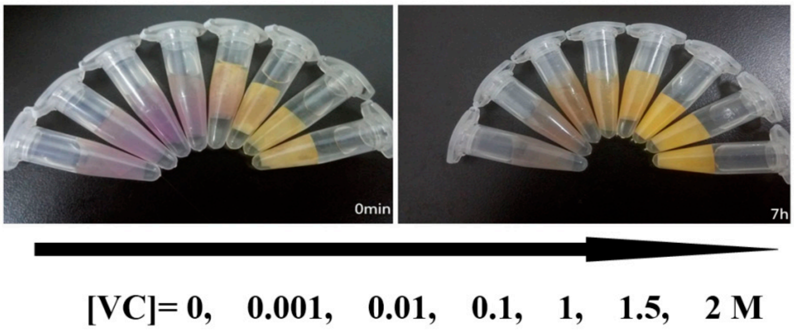

(**b**)

Figure S17. Influence of VC concentration on the preparation of Cu NCs@ESM at room temperature. (**a**) ESMs were pre-incubated in 1mL $CuSO_4$ (50 mM) for 10 min, then treated with 10 M NaOH solution for 30 min, and finally transferred to VC solutions with various concentrations (0, 0.001, 0.01, 0.05, 1, 1.5, 2 M, from left to right tubes). (**b**) Photoes of the samples at 0 min and after reactions of 7 hours under room light.

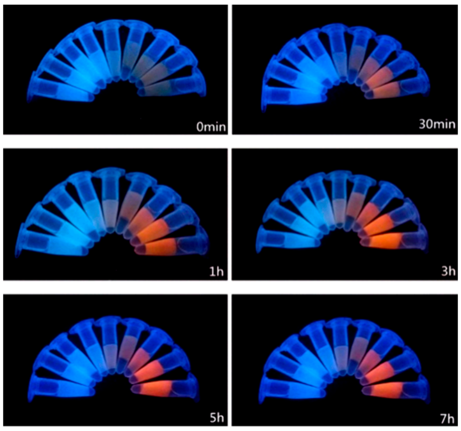

(**a**)

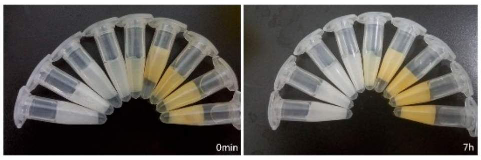

(**b**)

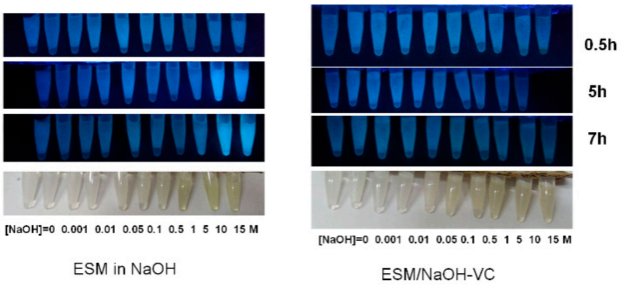

(**c**)

Figure S18. Influence of NaOH concentration on the generation of Cu NCs@ESM reduced by VC at room temperature. (**a**) ESMs were pre-incubated in 1mL $CuSO_4$ (50 mM) for 10min, then treated with NaOH aqueous solution of different concentrations (0, 0.001, 0.01, 0.1, 0.5, 1, 5, 10, 15 M, from left to right tubes) for 30 min, and finally transferred to 2 M VC solution. (**b**) Photos of the samples at 0min and after reactions of 7 hours under room light. (**c**) ESMs incubated in NaOH solutions with different concentrations (left); ESMs incubated in NaOH solutions with different concentrations for 30 min and then thrown into 1 mL 2 M VC solution (right).

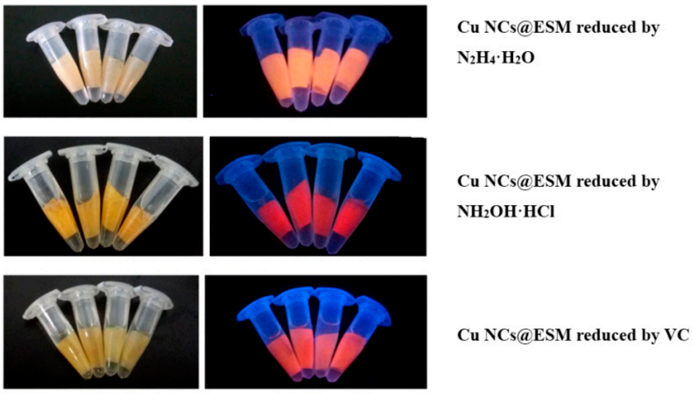

Figure S19. The effect of different copper precursors on the generation of fluorescent Cu NCs@ESM in the different three strategies. The various copper precursors such as $CuCl_2$, $Cu(NO_3)_2$, $Cu(CH_3COO)_2$, $CuSO_4$ were employed (from left to right tubes) in the strategies reduced by $N_2H_4{\cdot}H_2O$ (upper panels), $NH_2OH{\cdot}HCl$ (middle panels) and VC (lower panels). Photos of the samples under room light (left) and 365 nm UV light (right), respectively.

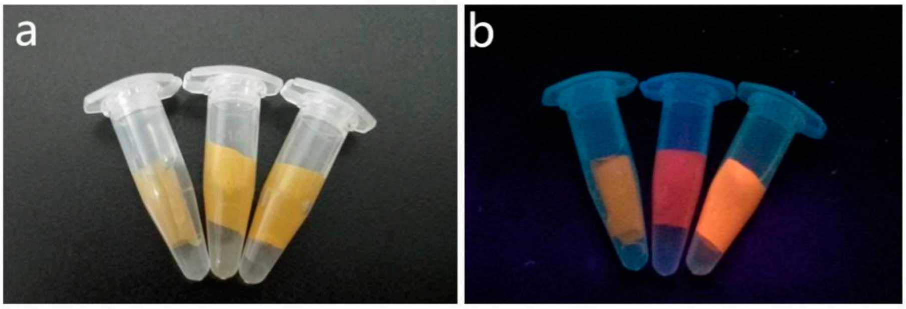

Figure S20. Photos of obtained Cu NCs@ESM generated by the three different synthetic routes employing boiled ESM platform under room light (**a**) and 365 nm UV light (**b**).

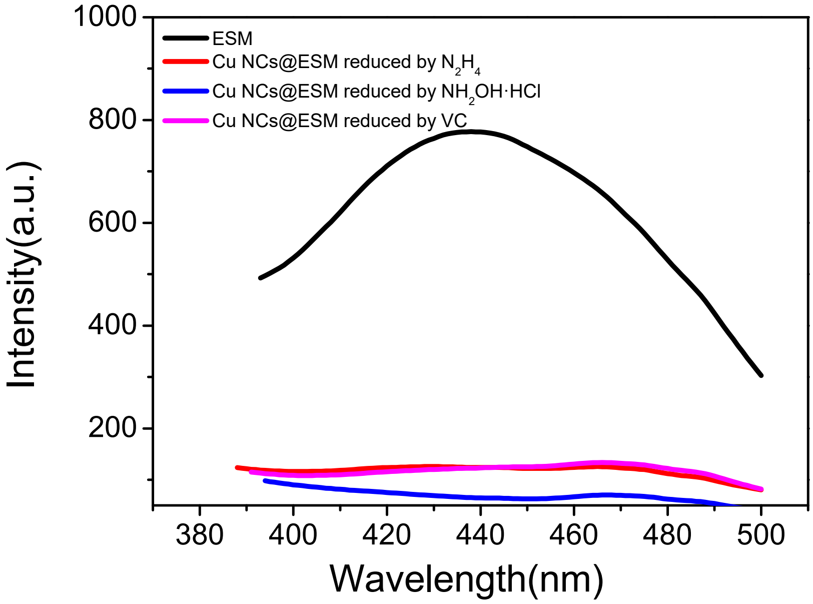

Figure S21. The fluorescence emissions (λ_{ex} = 365 nm) of ESM platforms from natural ESM (black line) and ESMs embedded with fluorescent Cu NCs in situ generated by $N_2H_4{\cdot}H_2O$ reduction (red line), $NH_2OH{\cdot}HCl$ reduction (blue line) and VC reduction (purple line), respectively.

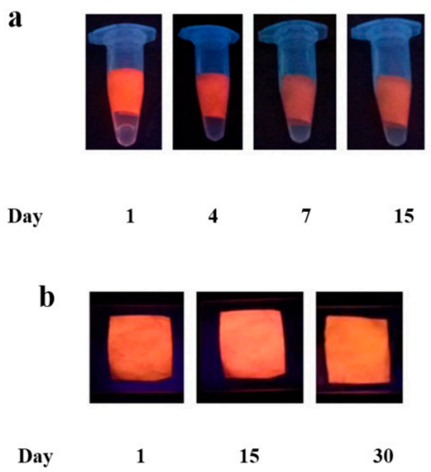

Figure S22. The stability of monolithic Cu NCs@ESM generated by VC reduction under alkaline condition stored in wet (**a**) and dried (**b**) forms stored at 4 °C in the refrigerator.

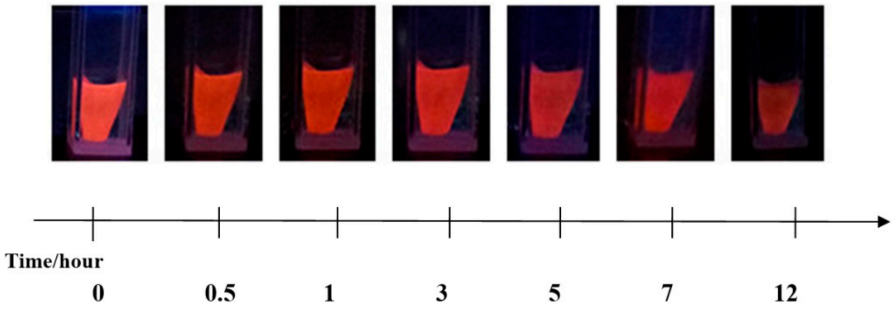

Figure S23. Photostability of the as-prepared Cu NCs@ESM evaluated by placing it in quartz cuvette under the 365 nm UV light irradiation from a 6 W mercury lamp with a distance of about 1 cm to the

lamp tube. Photos were taken after continuous irradiation of 0, 0.5, 1, 3, 5, 7, 12 hours respectively (from left to right).

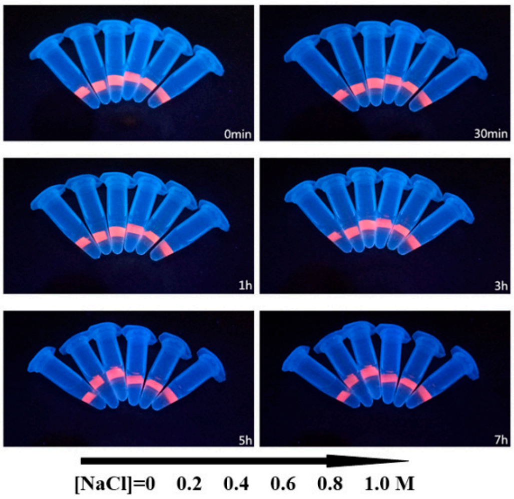

Figure S24. Salt tolerance of prepared Cu NCs@ESM in NaCl aqueous solutions with different concentrations (0, 0.2, 0.4, 0.6, 0.8, 1.0 M, from left to right).

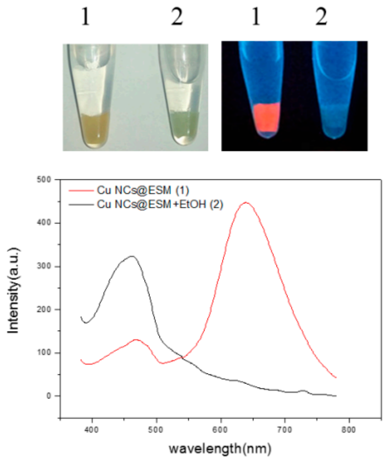

(**a**)

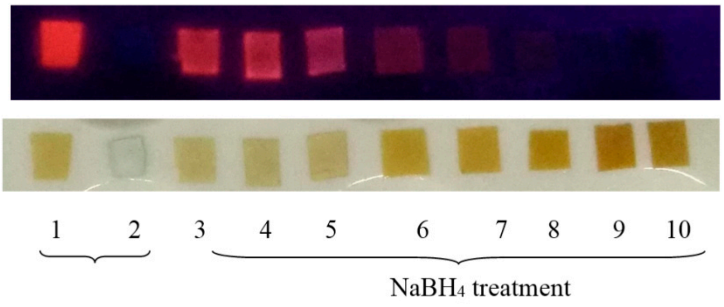

(**b**)

Figure S25. (**a**) The photos and fluorescence emission spectra of Cu NCs@ESM in the absence (1) and presence (2) of ethanol under room light (left) and the 365 nm UV light (right). (**b**) The fluorescence recovery experiment of quenched Cu NCs@ESM composite by $NaBH_4$ treatment. The quenched Cu NCs@ESMs were incubated with $NaBH_4$ aqueous solutions with different concentrations (500, 250, 125, 50, 25, 10, 5, 2.5 mM, from 3 to 10) to regenerate Cu NCs at room temperature for 2 hours.

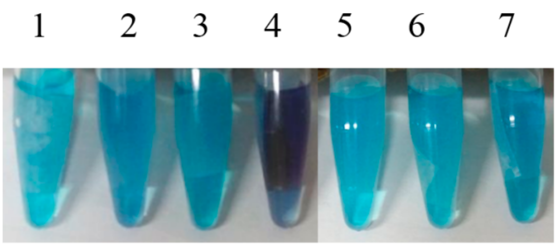

(**a**)

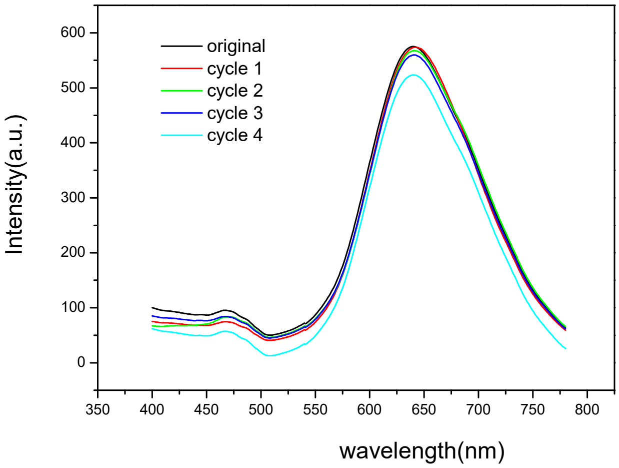

(**b**)

Figure S26. (**a**) The photo of MB aqueous solutions by adding serial control ESMs treated with the same way, respectively. (From 1 to 7: ESM only, ESM-NaOH, ESM-$CuSO_4$, ESM-$CuSO_4$-NaOH, ESM-$CuSO_4$-VC, ESM-NaOH-VC, ESM-VC.) (**b**) The fluorescence emission spectra of Cu NCs@ESM composite before and after catalysis excited by 365 nm.

© 2018 by the authors. Submitted for possible open access publication under the terms and conditions of the Creative Commons Attribution (CC BY) license (http://creativecommons.org/licenses/by/4.0/).
